# Chemodrug delivery using integrin-targeted PLGA-Chitosan nanoparticle for lung cancer therapy

**DOI:** 10.1038/s41598-017-15012-5

**Published:** 2017-11-07

**Authors:** Anish Babu, Narsireddy Amreddy, Ranganayaki Muralidharan, Gopal Pathuri, Hariprasad Gali, Allshine Chen, Yan D. Zhao, Anupama Munshi, Rajagopal Ramesh

**Affiliations:** 1 Department of Pathology, The University of Oklahoma Health Sciences Center, Oklahoma City, Oklahoma 73104 USA; 2 Department of Pharmaceutical Sciences, The University of Oklahoma Health Sciences Center, Oklahoma City, Oklahoma 73104 USA; 3 Department of Biostatistics and Epidemiology, The University of Oklahoma Health Sciences Center, Oklahoma City, Oklahoma 73104 USA; 4 Department of Radiation Oncology, The University of Oklahoma Health Sciences Center, Oklahoma City, Oklahoma 73104 USA; 5 Department of Medicine, The University of Oklahoma Health Sciences Center, Oklahoma City, Oklahoma 73104 USA; 6 Stephenson Cancer Center, The University of Oklahoma Health Sciences Center, Oklahoma City, Oklahoma 73104 USA; 7 Graduate Program in Biomedical Sciences, The University of Oklahoma Health Sciences Center, Oklahoma City, Oklahoma 73104 USA

## Abstract

In this study, we report the efficacy of RGD (arginine-glycine-aspartic acid) peptide-modified polylactic acid-co-glycolic acid (PLGA)-Chitosan nanoparticle (CSNP) for integrin α_v_β_3_ receptor targeted paclitaxel (PTX) delivery in lung cancer cells and its impact on normal cells. RGD peptide-modified chitosan was synthesized and then coated onto PTX-PLGA nanoparticles prepared by emulsion-solvent evaporation. PTX-PLGA-CSNP-RGD displayed favorable physicochemical properties for a targeted drug delivery system. The PTX-PLGA-CSNP-RGD system showed increased uptake *via* integrin receptor mediated endocytosis, triggered enhanced apoptosis, and induced G2/M cell cycle arrest and more overall cytotoxicity than its non-targeted counterpart in cancer cells. PTX-PLGA-CSNP-RGD showed less toxicity in lung fibroblasts than in cancer cells, may be attributed to low drug sensitivity, nevertheless the study invited close attention to their transient overexpression of integrin α_v_β_3_ and cautioned against corresponding uptake of toxic drugs, if any at all. Whereas, normal human bronchial epithelial (NHBE) cells with poor integrin α_v_β_3_ expression showed negligible toxicity to PTX-PLGA-CSNP-RGD, at equivalent drug concentrations used in cancer cells. Further, the nanoparticle demonstrated its capacity in targeted delivery of Cisplatin (CDDP), a drug having physicochemical properties different to PTX. Taken together, our study demonstrates that PLGA-CSNP-RGD is a promising nanoplatform for integrin targeted chemotherapeutic delivery to lung cancer.

## Introduction

Since most chemotherapeutic drugs are toxic to normal cells, achieving the relevant therapeutic drug concentration in cancer cells while reducing systemic exposure to the drug is an important goal^1–4^. The non-specific, primarily dose-dependent toxicity of chemotherapeutics toward normal cells is a continuing problem. However, targeted nanoparticle-based drug delivery is a highly promising strategy to overcome this challenge^5,6^. Targeted drug delivery systems show higher affinity toward tumor cells overexpressing specific receptors than toward normal cells^7,8^.

In lung cancers, the overexpression of cell-surface receptors is often exploited for targeted delivery of therapeutics with ligand−/antibody-modified nano-drug delivery vehicles^9,10^. The integrin (α_v_β_3_) receptor is of particular interest, since its expression is high in tumor endothelium and tumor cells^11,12^. Using Arg-Gly-Asp (RGD) peptide to target integrin (α_v_β_3_) in tumor vascular endothelium is a well-known strategy to suppress angiogenesis and metastasis^11,13–15^. The specific affinity of RGD sequence and integrin (α_v_β_3_) has also been harnessed for targeted drug delivery^16,17^ and diagnostic applications using nanoparticles^18,19^. The expression of integrins is relatively weak in normal cells. However, transient overexpression of integrins are observed in some normal cell lines including lung fibroblasts, although at variable levels^20,21^. Therefore, while highlighting the integrin receptor targeted-nanoparticle based drug delivery in cancer cells, it is also important to consider the impact of targeted drug delivery in normal cells that exhibit high level of integrin receptor expression. Based on these reports we hypothesized that RGD modified nanoparticles will preferentially target and deliver chemodrugs to integrin receptor overexpressing lung cancer cells and produce increased therapeutic efficiency while sparing integrin non-expressing normal cells from the drug toxicity.

Herein, we designed an RGD modified poly-lactic-acid-co-glycolic acid (PLGA)-chitosan-based nanoparticle system (PLGA-CSNP-RGD) for targeted drug delivery in non-small cell lung carcinoma (NSCLC) cells having high levels of α_v_β_3_ integrin expression. The nanoparticle system has a PLGA core loaded with drug, and is surface-coated with chitosan, to which linear RGD peptide (GRGDSP) is conjugated. Chitosan, a biocompatible cationic polymer, possesses numerous functional groups for targeting ligand modification^22^. Moreover, chitosan coating enhances the particle stability and controls drug release^23^. Chitosan’s muco-adhesive property can be exploited for trans-mucosal delivery of drugs, especially through the intrapulmonary route^24^. In addition, GRGDSP is a linear peptide that preferentially recognizes the integrin α_v_β_3_^25^ and α_5_β_1_ receptors expressed on the cell surface^26^. The cell adhesion capacity of GRGDSP peptide is several times higher than similar peptides that have affinity towards fibronectin receptors^27^. Studies also have shown that GRGDSP peptide-functionalized nanoparticles possess excellent cell-adhesion properties *via* integrin receptors and are being used for targeted delivery of drugs and diagnostic agents^28–32^. These advantages of RGD peptide, chitosan and PLGA nanoparticle have been integrated in our novel formulation for integrin-targeted drug delivery in lung cancer cells.

We tested this PLGA-CSNP-RGD system in NSCLC cells overexpressing integrin α_v_β_3_ receptors. First, we used western blot analysis and flow cytometry to examine the integrin α_v_β_3_ expression levels in a panel of NSCLC cells and normal cells. Then, the targeted nanoparticle was loaded with paclitaxel (PTX), a potent anti-cancer drug, and cell-killing efficiency of this targeted nanoparticle was compared with that of free PTX and non-targeted nanoparticles. Apoptosis and cell cycle analysis were performed to confirm the therapeutic activity. Then, the efficiency of PLGA-CSNP-RGD was tested in different NSCLC cell lines and normal cells with different levels of integrin expression. Differential toxicity of PTX-PLGA-CSNP-RGD was confirmed in NSCLC and normal lung fibroblasts, while broncho-epithelial cells showed negligible response to the toxicity of PTX delivered using PLGA-CSNP-RGD. Finally, we confirmed the potential of PLGA-CSNP-RGD as a delivery platform for an alternative drug cisplatin (CDDP), a widely-used drug in lung cancer therapy.

## Results and Discussion

### Baseline expression of integrin α_v_β_3_ receptors

**Figure 1:**
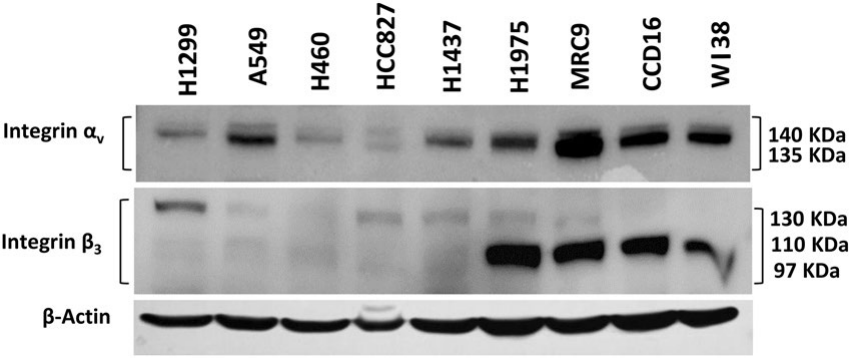
Baseline expression of integrin α_v_β_3_ in a panel of lung cancer (H1299, A549, H460, HCC827, H1437, H1975) and lung fibroblast (MRC9, CCD16, WI38) cell lines. Expression of integrin α_v_ and actin was determined from one gel and integrin β_3_ was determined from a separate gel.

The baseline expression levels of integrin α_v_β_3_ receptor in various lung cancer cell lines and lung fibroblasts were determined by Western blotting [Fig. 1, Table ST1] and by flow cytometric analysis [Figure S1]. Among the lung cancer cell lines the highest integrin α_v_β_3_ receptor expression was observed in H1975. All the lung fibroblasts studied showed high expressions levels of integrin α_v_β_3_, among which MRC-9 showed the highest expression. From the lung cancer panel we have chosen H1975, A549 and H1299, and from the normal lung fibroblasts we have selected MRC-9, respectively based on their integrin α_v_β_3_ expression levels, for our studies. We believed that it would be of interest to know the effect of integrin α_v_β_3_ receptor targeted delivery of nanoparticles carrying chemotherapeutics in selectivity and cytotoxicity in lung cancer and fibroblast cells with high integrin α_v_β_3_ receptor expressions.

### Preparation and characterization of nanoparticles

Preparation of drug-loaded PLGA-CSNP-RGD nanoparticles has three distinctive phases: 1) synthesis of PTX-loaded PLGA nanoparticles using the emulsion-solvent evaporation technique, 2) synthesis of RGD-peptide-modified chitosan, and 3) coating RGD-CS using the deposition method to prepare drug-loaded PLGA-CSNP-RGD. PLGA nanoparticles can encapsulate hydrophobic^33^ or hydrophilic drugs^34^ based on the method used for the preparation. Particle sizes, zeta potentials, and polydispersity indexes of the nanoparticles were different in each stage of nanoparticle development. As shown in Table 1, the average hydrodynamic size, measured as intensity versus diameter, of the PTX-PLGANP was 175 nm, and the RGD-chitosan modification of PTX-PLGANP resulted in 217 nm particles, an average increase of 42 nm contributed by CS-RGD. The strong negative surface charge of acid-terminated PLGA was utilized to deposit positively charged CS as a coating onto PLGANP. Thus, the negative zeta potential of PTX-PLGANP becomes positive upon CS coating, which slightly reduced in the presence of RGD peptide in chitosan polymer. One of the major reasons that we have used chitosan as a coating material for PLGA nanoparticle is to explore the presence of numerous free amino groups for functionalization, for instance here we have used this property for RGD peptide modification. Studies also suggest that chitosan coating via physical adsorption is a successful strategy used for controlled release of drugs and enhance cell uptake of PLGA based nanoparticles^35–37^. Moreover, chitosan coating also provides a cationic layer that can electrostatically bind to negatively charged therapeutic molecules such as nucleic acid therapeutics. In fact, our previous study proved that successful co-delivery of siRNA/pDNA with chemotherapeutics is possible using chitosan coated poly-lactic-acid nanoparticles^38^.

**Table 1:** Size, charge and dispersion values of PTX-nanoformulations.

	Particle Size (d.nm)	PDI	Zeta potential (mV)
PTX-PLGANP	175.25 ± 6.55	0.088 ± 0.006	(−)25.25 ± 3.3
PTX-PLGA-CSNP	212.5 ± 6.02	0.140 ± 0.023 2	(+)33.4 ± 0.8
PTX-PLGA-CSNP-RGD	217 ± 13.54	0.133 ± 0.011	(+)29.5 ± 4.04

*(n = 4).

**Figure 2:**
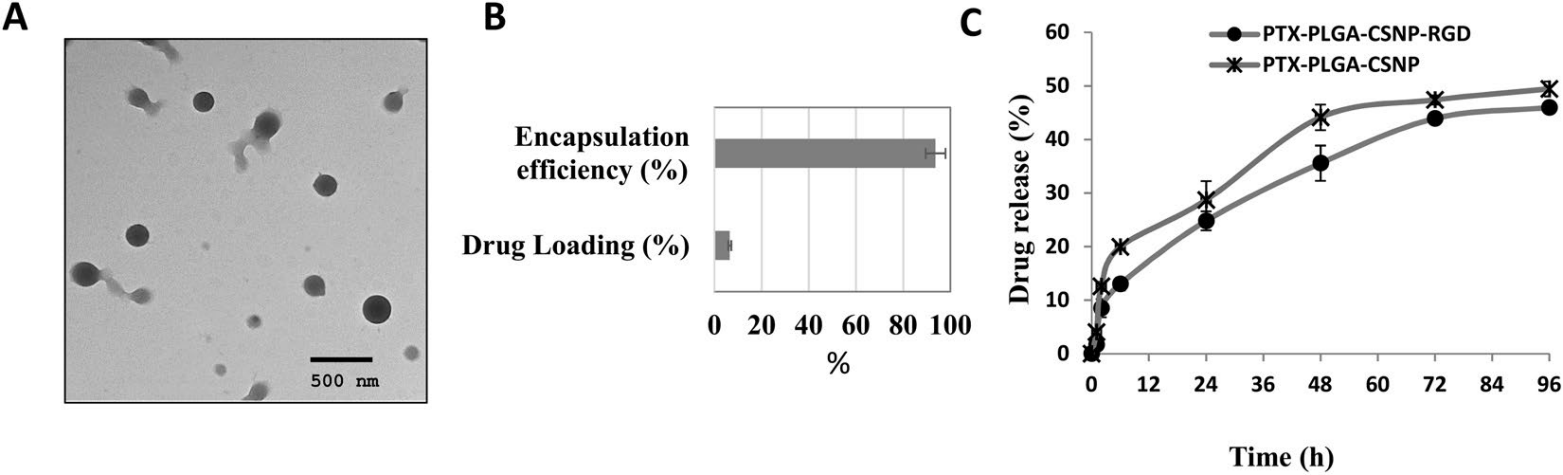
Physicochemical characterization of PTX-PLGA-CSNP-RGD. (**A**) TEM images of PTX-PLGA-CSNP-RGD; (**B**) Drug loading and encapsulation efficiencies of PTX-PLGA-CSNP-RGD. (n = 5); (**C**) *In vitro* drug (PTX) release from unmodified nanoparticles (PTX-PLGA-CSNP) and RGD modified nanoparticles (PTX-PLGA-CSNP-RGD) in PBS pH 7.4.

We used maleimide crosslinking chemistry to conjugate GRGDSP peptide with chitosan polymer. The multiple steps involved in the synthesis of RGD-CS are depicted in Figure S2. The PEG polymer present in the maleimide linker might be useful in preventing rapid bio-clearance of nanoparticles^39^. The PTX-PLGA-CSNP-RGD particles were well dispersed and had spherical morphology, as shown in the representative TEM image [Fig. 2A]. The final particles (PTX-PLGA-CSNP-RGD) were stable in aqueous solution in 4 °C for at least two weeks, without any visible sedimentation but with less than 5% increase in particle size as measured by DLS [Figure S3]. However, when we incubated the nanoparticles with different serum concentrations slight to significant increase in particle sizes and change in zeta potentials were observed, depending on serum concentrations and time of incubation [Table ST2]. This indicates the formation of biocorona in nanoparticles. When A549 cells were treated with these sera incubated nanoparticles (FluTax-PLGA-CSNP-RGD), we observed that cell accumulation of the fluorescent paclitaxel depended on serum concentrations [Figure S4]. Typically, low serum concentrations (2% and 5%) did not have any impact on cell uptake while 10% serum reduced the cell uptake of FluTax-PLGA-CSNP-RGD (*p* < 0.05). While it is known that protein corona formation is influenced by physicochemical properties of the nanoparticles, reports suggest that the biological fate of nanoparticle is however determined by the identity of the protein corona rather than the changes in physicochemical properties induced by corona formation^40,41^.

Further, PLGA-CSNP-RGD showed an average PTX encapsulation efficiency of 93.7% and drug loading of 6.5% [Fig. 2B]. The drug loading efficiency of our nanoparticle seems reasonable and is consistent with range of drug loading found in literature, from 1% wt/wt^42^ to 10.46% wt/wt^43^ or higher, that have shown successful therapeutic efficiency.

Next, we measured the drug release profile from RGD modified (PTX-PLGA-CSNP-RGD) and unmodified (PTX- PLGA-CSNP) nanoparticles in PBS (pH 7.4) [Fig. 2C]. At 1 h an average 4% of PTX was released from RGD unmodified system (PTX-PLGA-CSNP) compared only 1% from RGD modified nanoparticles (PTX-PLGA-CSNP-RGD; *p* < 0.05). In the first 6 h, around 13% PTX was released from PTX-PLGA-CSNP-RGD compared to 19.9% from PTX-PLGA-CSNP, suggesting a moderate burst release. At the early time points, drug release rate might have influenced by initial thrust in the water penetration through the nanoparticle matrix^44^. However, the release of PTX was slowed down over time; as observed over 24 h through 96 h the drug release reached 24.8% and 45.9%, respectively for PTX-PLGA-CSNP-RGD. The PTX release in PTX-PLGA-CSNP also followed a similar pattern observed with PTX-PLGA-CSNP-RGD (P > 0.05), showing no significant difference in 24 h (28.7%) though 96 h (49.4%). Therefore, our results clearly indicate that the drug release pattern is similar for both unmodified and modified nanoparticles. The core PLGA matrix with CS or CS-RGD modification might have controlled and sustained the diffusion of PTX over time. However, there are many complex variables that influence the drug release pattern such as drug properties and affinity of drug towards the polymer matrix, polymer composition, polymer degradation and erosion rate, temperature, pH, release medium type and/or a combination of these processes^44,45^. Nevertheless, our results are consistent with the previous reports suggesting that the release of hydrophobic drugs from PLA or PLGA nanoparticles occurs slowly and over several days^46^.

Another important point is that we measured the drug remained in the nanoparticles in the dialysis apparatus (retained drug) rather than in the releasing medium^47^. However, the retained drug quantity was calculated for all the time points of study in three separate experiments. This method was followed to avoid the analytical limitation that we had with the HPLC measurement. Further, our study was focused to understand whether the drug release could be controlled and released over time using our nanoparticles, which we could observe by following drug release up to 96 h. However, based on the release profile observed the release might continue for several days after 96 h. The drug release is thus predicted to be in the same fashion continued for a longer period as reported in similar systems in the literature^48,49^.

### Optimization of RGD concentration for efficient cell uptake of nanoparticles

For enhanced nanoparticle uptake by receptor-overexpressing cancer cells, nanoparticles should present an optimal ligand concentration on their surfaces^50^. We investigated the targeting potential of Rhodamine B-encapsulated-PLGA-CSNP modified with various concentrations of RGD in A549 lung cancer cells. The nanoparticles were incubated with cells for 6 h and 24 h. Figure S5A and B shows the fluorescent microscopy images and fluorescence intensity plot respectively from the Envision microplate reading experiment. The highest cell uptake was observed with RB-PLGA-CSNP with 0.34 µM RGD concentration. At 6 h and 24 h the average fluorescence intensities of 0.34 µM RGD group was 96942.67 (a.u.) and 226756.66 (a.u.) respectively. These values were significantly higher than untreated control and other groups with different RGD concentrations, especially at 6 h (*p* < 0.01). This observation from fluorescence intensity measurements was supported by microscopy images that showed the elevated RB fluorescence at 6 h and 24 h, obtained from RB-PLGA-CSNP-treated groups. Henceforth, all our experiments were conducted with this RGD (0.34 µM) concentration in PLGA-CSNP.

### Cell uptake of targeted versus non-targeted nanoparticles

**Figure 3:**
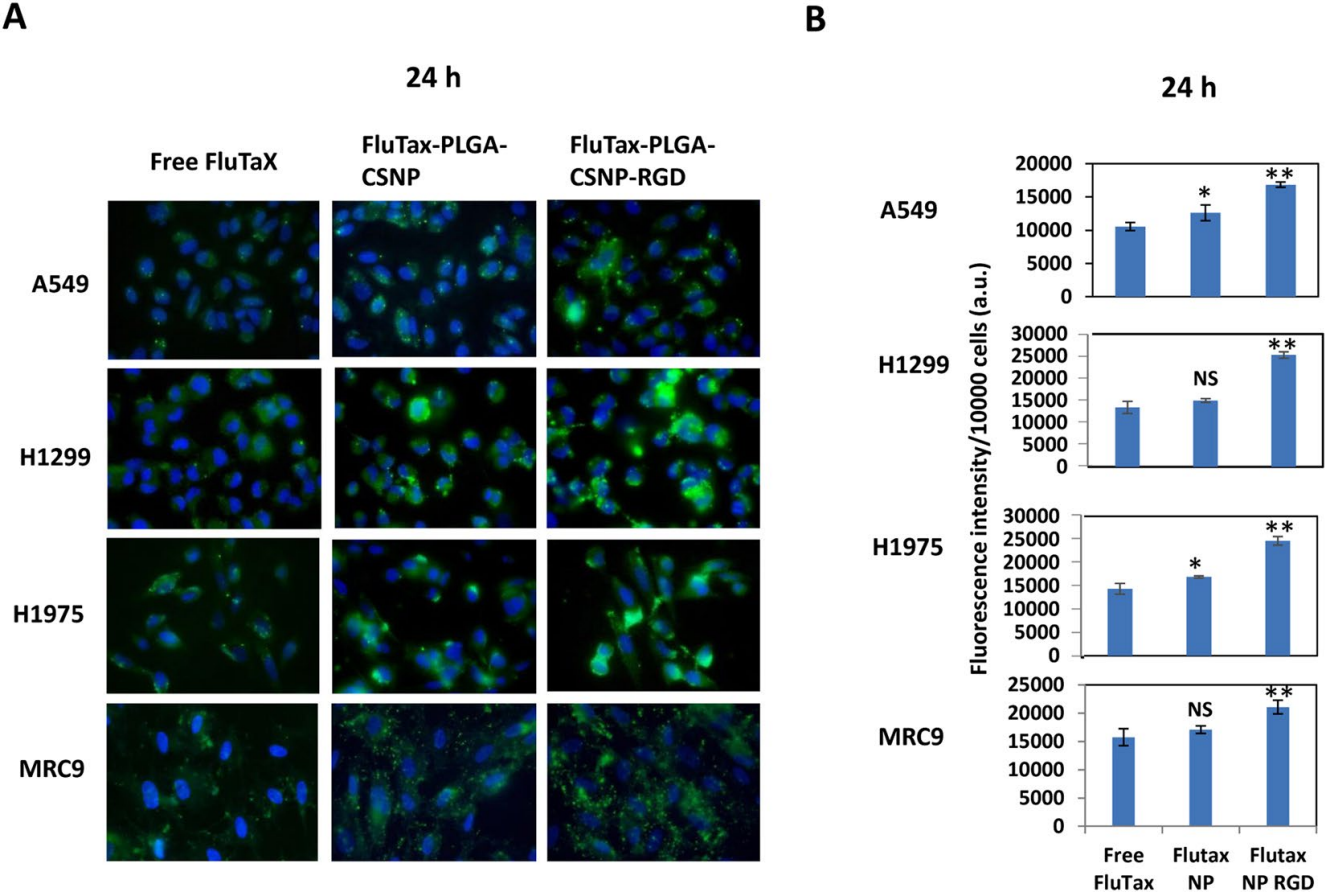
Cell uptake studies. (**A**) Fluorescent microscopy images and, (**B**) graphical representation of fluorescence intensity per 10000 cells of uptake of Free Flutax (Fluorescent Taxol-Oregon Green 488), and FluTax-PLGA-CSNP with or without RGD modification in A549, H1299, H1975 and MRC-9 cells for 24 h. The studies for each cell line were performed separately and the photos assembled to represent the treatment group for each cell line. *Magnification* 60X.

Integrin α_v_β_3_ expression levels are different for different lung cancer cell lines^51^. Therefore, the RGD peptide-based selective uptake of nanoparticles may depend on integrin α_v_β_3_ expression levels. To understand the cell uptake of nanoparticles in lung cancer cells and normal cells, we incubated the cells with fluorescent paclitaxel Oregon green 488 (FluTax)-PLGA-CSNP, with or without RGD modification. Figures 3A,B and S6 shows the fluorescent images and graphical representation of fluorescence intensity corresponding to FluTax obtained from Free Flutax and FluTax-PLGA-CSNP-(RGD+/−) treated cells. The results clearly show that targeted (RGD-modified) nanoparticles showed enhanced FluTax delivery compared with non-targeted (unmodified) nanoparticles and Free FluTax. Notably, FluTax fluorescence corresponded to integrin α_v_β_3_ expression levels in lung cancer cells, when considering the nanoparticle delivery groups. Free FluTax, irrespective of cell lines and time points, showed cell uptake which is either comparable or lesser than non-targeted nanoparticle groups (FluTax-PLGA-CSNP), but significantly lesser uptake than targeted nanoparticle groups (FluTax-PLGA-CSNP-RGD) (*p* < 0.01). Based on the fluorescence intensity obtained from fluorescent paclitaxel (FluTax), 2 nM FluTax equivalent treatment of A549 cells using FluTax-PLGA-CSNP-RGD resulted in an uptake of 28 pg/1 × 10^5^ cells in 24 h. In contrast free PTX uptake was 15.5 pg/1 × 10^5^ cells, and for non-targeted nanoparticles it was 20 pg/1 × 10^5^ cells. This was calculated from fluorescence intensity of known FluTax concentration (2 nM, 100 ul), kept as positive control.

Binding of RGD peptide-integrin α_v_β_3_ receptors affinity mediates the endocytotic uptake of RGD-modified nanoparticle^52^. Among NSCLC cells, H1975 cells displayed the highest uptake of RGD-modified nanoparticles, an expected result based on their higher integrin α_v_β_3_ expression. Based on their expression of integrin α_v_β_3_, we anticipated that MRC-9 cells would display enhanced uptake of RGD-modified nanoparticles, as observed in the present study. However, normal bronchial epithelial cells (NHBE), with negligible integrin α_v_β_3_ expression, showed the least uptake of RGD-modified nanoparticles [Figure S7A and B]. This finding clearly suggests that uptake of FluTax-PLGA-CSNP-RGD was dependent on integrin α_v_β_3_ expression levels in cells, which took up more FluTax-PLGA-CSNP-RGD than unmodified FluTaX-PLGA-CSNP. However, unmodified nanoparticles with positive charge may efficiently interact with negatively charged cell membrane; as a result cellular entry is possible by charge-mediated adsorptive endocytosis^53^. Such a cellular entry mechanism for cationic particles is non-specific that does not differentiate between cancer cells and normal cells. Interestingly, decoration of nanoparticle using positively charged chitosan itself was reported to have selectivity towards cancer cells compared to normal cells, since cancer cells showed a stronger net-negative charge on the cell membrane^54^. Therefore, a combined effect of charge based interaction and RGD based specific targeting might have contributed to the observed cell accumulation FluTax-PLGA-CSNP-RGD. Nevertheless the cell uptake study highlights that RGD conjugation selectively increased the targeting of the PTX-PLGA-CSNP to lung cancer cells, but not to normal broncho-epithelial cells. Since MRC-9 cells showed selective uptake of FluTax-PLGA-CSNP-RGD, we further wanted to determine whether this cell uptake translated to cytotoxicity of delivered drug.

### Cell killing efficiency of PTX formulations compared with free PTX in NSCLC cells

**Figure 4:**
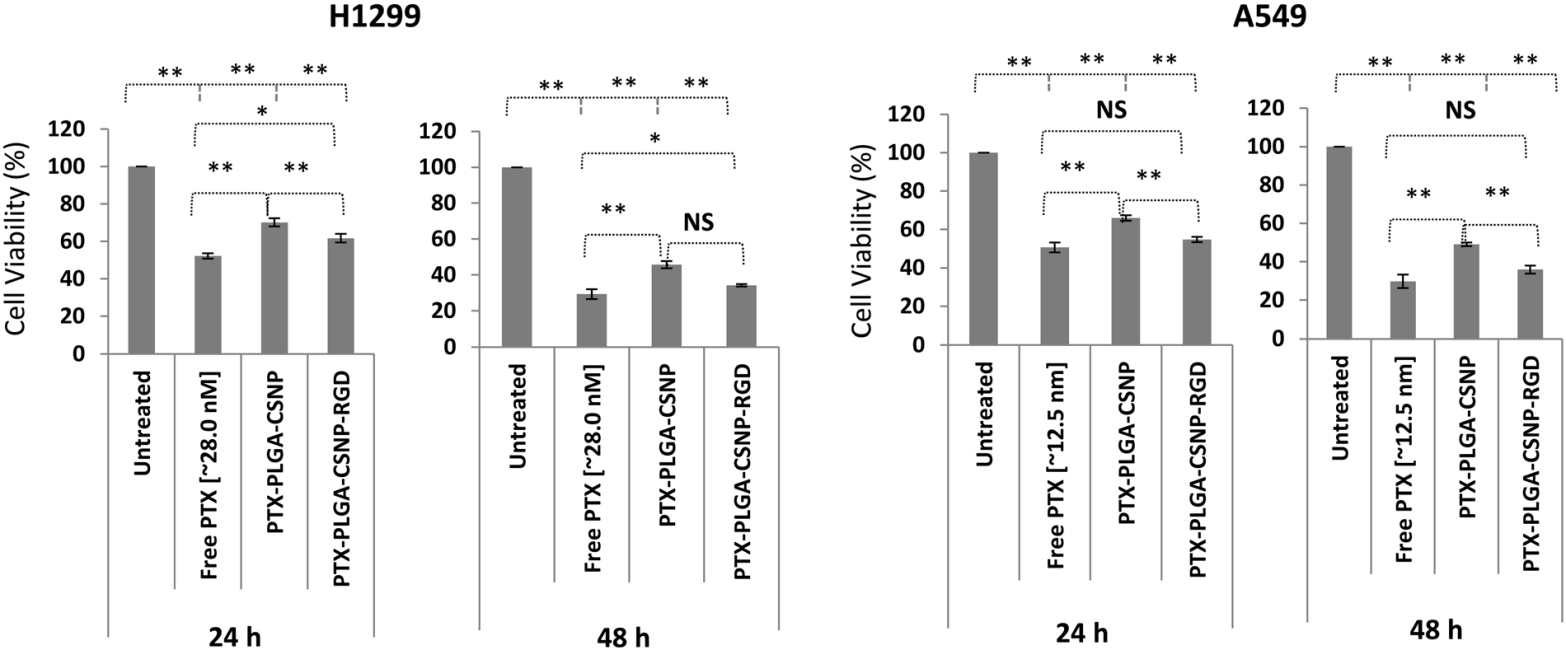
Comparative cell killing efficiency of PTX formulations in NSCLC cells. Cell viability (%) of H1299 and A549 cells when treated with free PTX, PTX-PLGA-CSNP, and PTX-PLGA-CSNP-RGD for 24 h and 48 h measured by trypan blue exclusion assay. (n = 3); *p < 0.05; **p < 0.01; NS, non-significant.

To evaluate the cell killing efficiency of free-PTX and PTX formulations, cell viability experiments were conducted for 24 h and 48 h post-incubation. The IC_50_ values in A549 (~12.5 nM) and H1299 (~28.0 nM) obtained from a standard logarithmic plot [Figure S8] were chosen as the PTX doses for our cell viability experiment to compare free PTX and PTX-PGA-CSNP-(RGD+/−) formulations. As shown in Fig. 4, free PTX showed the highest cytotoxicity in A549 and H1299 cell lines at both time points. The cell killing efficiency of PTX-PLGA-CSNP was moderate at equivalent PTX doses compared with free PTX. This trend was expected, as the PTX release from nanoparticles is slow and controlled over time. We observed that the average (%) cell viability of free PTX and PTX-PLGA-CSNP-RGD treatment groups in A549 cells was comparable at 24 h (50.6% and 54.7% respectively, *p* > 0.05) and 48 h (29.7% and 35.9% respectively, *p* > 0.05), whereas free PTX showed significant toxicity compared with PTX-PLGA-CSNP-RGD in H1299 cells (*p* < 0.05).

However, PTX-PLGA-CSNP-RGD showed reduced cell viability in both cell lines, compared with PTX-PLGA-CSNP. For instance, the margin of cell viability (%) between PTX-PLGA-CSNP-treated A549 cells and PTX-PLGA-CSNP-RGD-treated A549 cells was significant at 24 h (65.9% and 54.7%, respectively) (*p* < 0.01) and 48 h (49.1% and 35.9%) (*p* < 0.01) indicating a clear enhanced cell killing efficiency of PTX-PLGA-CSNP-RGD treatment. H1299 cells also showed a similar trend in cell viability efficacy of PTX-PLGA-CSNP-RGD compared with PTX-PLGA-CSNP, especially at 24 h. The cell viability for PTX-PLGA-CSNP group was 70.1%, which is significantly higher than PTX-PLGA-CSNP-RGD group with 61.6% (*p* < 0.01). However, this difference was not significant at 48 h. The cell viability for PTX-PLGA-CSNP group was 45.7%, which is significantly higher than PTX-PLGA-CSNP-RGD group with 35.2% (*p* < 0.01). The overall reduction in cell viability in PTX-PLGA-CSNP-RGD indicates that the accumulation of PTX is increased with integrin targeted delivery compared to non-targeted delivery, resulting in corresponding toxicity levels.

### PTX-PLGA-CSNP-RGD induces G2/M cell cycle arrest and significant apoptosis in NSCLC cells

**Figure 5:**
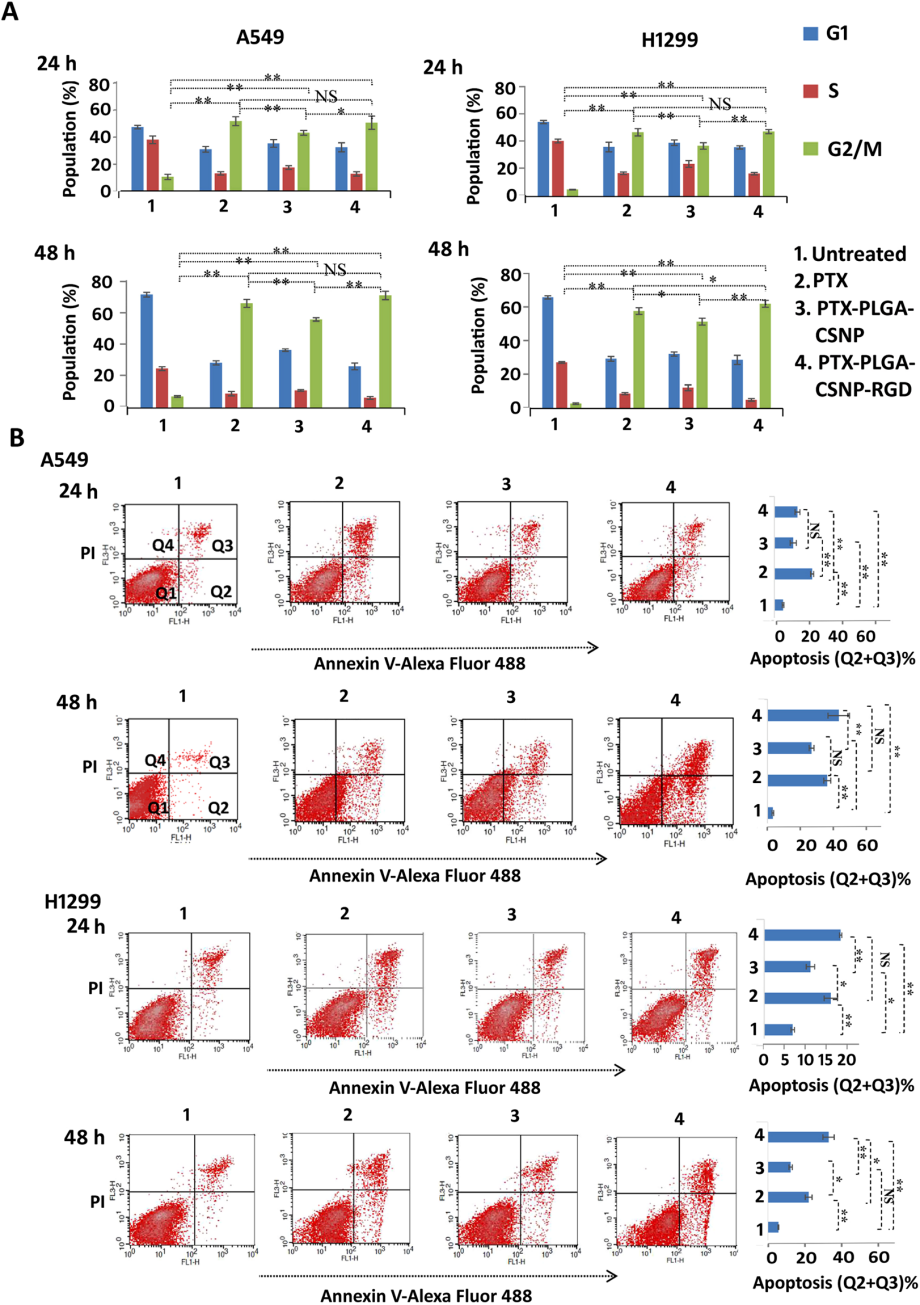
Cell cycle and apoptosis analysis in nanoparticle-treated lung tumor cells. (**A**) Graphical representation of percentage population of cells in G1, S and G2/M phases of cell cycle at 24 h and 48 h following treatment with treated with either 12.5 nM (A549) or 28.0 nM (H1299) equivalents of PTX in free, PTX-PLGA-CSNP or PTX-PLGA-CSNP-RGD formulations. (**B**) Dot plot (left) and graphical representations (right) of total apoptotic cells obtained by flow cytometry analysis of Annexin V- Alexa Fluor 488/PI stained A549 and H1299 cells at 24 h and 48 h time points. Cells were treated with either 12.5 nM (A549) or 28.0 nM (H1299) equivalents of PTX in free, PTX-PLGA-CSNP or PTX-PLGA-CSNP-RGD formulations. Total apoptotic cells (%) were the sum of quadrants Q2 and Q3. (n = 3); *p < 0.05, **p < 0.01; Statistical analysis was represented for G2 populations between treatment groups. (n = 3); *p < 0.05; **p < 0.01; NS, non-significant.

The effect of PTX, PTX-PLGA-CSNP, and PTX-PLGA-CSNP-RGD in cell cycle was analyzed in H1299 and A549 cells after 24 h and 48 h of treatment. Figure 5A shows the graphical representation of cell cycle phases (%) compared with the untreated controls. We observed that the free PTX and PTX-nanoparticles induced cell cycle arrest at the G2/M cell cycle. No significant differences were observed between free PTX and PTX-PLGA-CSNP-RGD formulations in either cell line at 24 h and 48 h. A comparatively smaller cell population showed G2/M cell cycle arrest with PTX-PLGA-CSNP treatment. In A549 cells at 24 h, the G2/M populations were 54.1%, 45%, and 52.9%, respectively for cells treated with free PTX, PTX-PLGA-CSNP, and PTX-PLGA-CSNP-RGD. Further, a time-dependent increase in G2/M arrest was observed in A549 cells at 48 h: 63.6%, 53.9%, and 68.5%, for free PTX, PTX-PLGA-CSNP, and PTX-PLGA-CSNP-RGD treatment groups respectively. The results showed a significant enhancement in G2/M populations because of PTX treatment irrespective of the formulations compared to untreated controls (*p* < 0.01). Similarly, in H1299 cells at 24 h, the G2/M populations were 46.8%, 36.9%, and 47.4%, respectively for cells treated with free PTX, PTX-PLGA-CSNP, and PTX-PLGA-CSNP-RGD. The PTX-PLGA-CSNP-RGD group induced significantly higher G2/M arrest compared to PTX-PLGA-CSNP group (*p* < 0.01). At 48 h H1299 cells showed 60.1%, 53.5%, and 64.6% G2/M populations (%), for free PTX, PTX-PLGA-CSNP, and PTX-PLGA-CSNP-RGD treatment groups respectively. The PTX-PLGA-CSNP-RGD treated group showed significantly higher G2/M cycle arrest compared to PTX (*p* < 0.05) and PTX-PLGA-CSNP (*p* < 0.01) respectively. Our result confirms that PLGA-CSNP-(RGD+/−)-assisted delivery of PTX induced apoptosis by the same cell cycle arrest mechanism as reported for free PTX^55^. The cell cycle arrest mechanism of PTX is dose-dependent, and the present dose falls within the range of PTX doses that produced similar cell cycle arrest effect (G2/M), as previously reported^56^. RGD-based targeting of PTX- PLGA-CSNP induced more G2/M arrest than did non-targeted delivery of PTX- PLGA-CSNP, demonstrating the superior efficiency of PTX- PLGA-CSNP-RGD in inducing apoptosis. Importantly, the previously observed Annexing/PI based apoptotic data corroborate the cell cycle results.

Since we observed that enhanced cytotoxicity and G2/M cell cycle arrest were induced by PTX-PLGA-CSNP-RGD compared PTX-PLGA-CSNP, we sought to quantify the apoptotic population further. Figure 5B shows the representative dot-plots of A549 and H1299 cells as measured by flow cytometry. Apoptosis was evident in PTX-, PTX-PLGA-CSNP-, and PTX-PLGA-CSNP-RGD-treated groups in both cell lines at −24 h and −48 h post-incubation. Live cells are represented in the lower left quadrant (Q1). Early apoptotic cells that stained positive only for Annexin V are plotted in the lower right quadrant (Q2). Cells that were positive for both Annexin V and PI are plotted in the upper right quadrant (Q3). The PI-only stained dead cell population is shown in the upper left quadrant (Q4). At 24 h, the total apoptotic cells (Q2 + Q3; %) in the PTX-PLGA-CSNP-RGD groups was comparable to that of the free PTX group in both H1299 and A549 cells. At 48 h, the PTX-PLGA-CSNP-RGD-treated A549 cells showed a significantly enhanced apoptotic population (41.86%) compared with the PTX-PLGA-CSNP-treated (25.84%; *p* < 0.01) cells, but not with the free-PTX-treated cells (35.04%; *p* > 0.05). H1299 cells also showed significantly enhanced apoptosis when treated with PTX-PLGA-CSNP-RGD compared with PTX-PLGA-CSNP (*p* < 0.01); however, this difference was not significant when compared with cells treated with free PTX. The total number of apoptotic cells in H1299 cells at 24 h followed the pattern PTX-PLGA-CSNP (10.92%) < PTX (15.74%) < PTX-PLGA-CSNP-RGD (18.96%). At 48 h PTX group showed 21.93% apoptotic cells, whereas PTX-PLGA-CSNP and PTX-PLGA-CSNP-RGD showed 12.24% and 32.54% apoptotic cells respectively. Overall, it is clear that PTX is capable of inducing apoptosis in its free form as well as nanoencapsulated forms. Moreover, these findings indicate that apoptosis induced by PTX-PLGA-CSNP-RGD is dependent on integrin expression levels in A549 and H1299 cells.

### Integrin α_v_β_3_ blocking revealed the targeting efficiency of PTX-PLGA-CSNP-RGD

**Figure 6:**
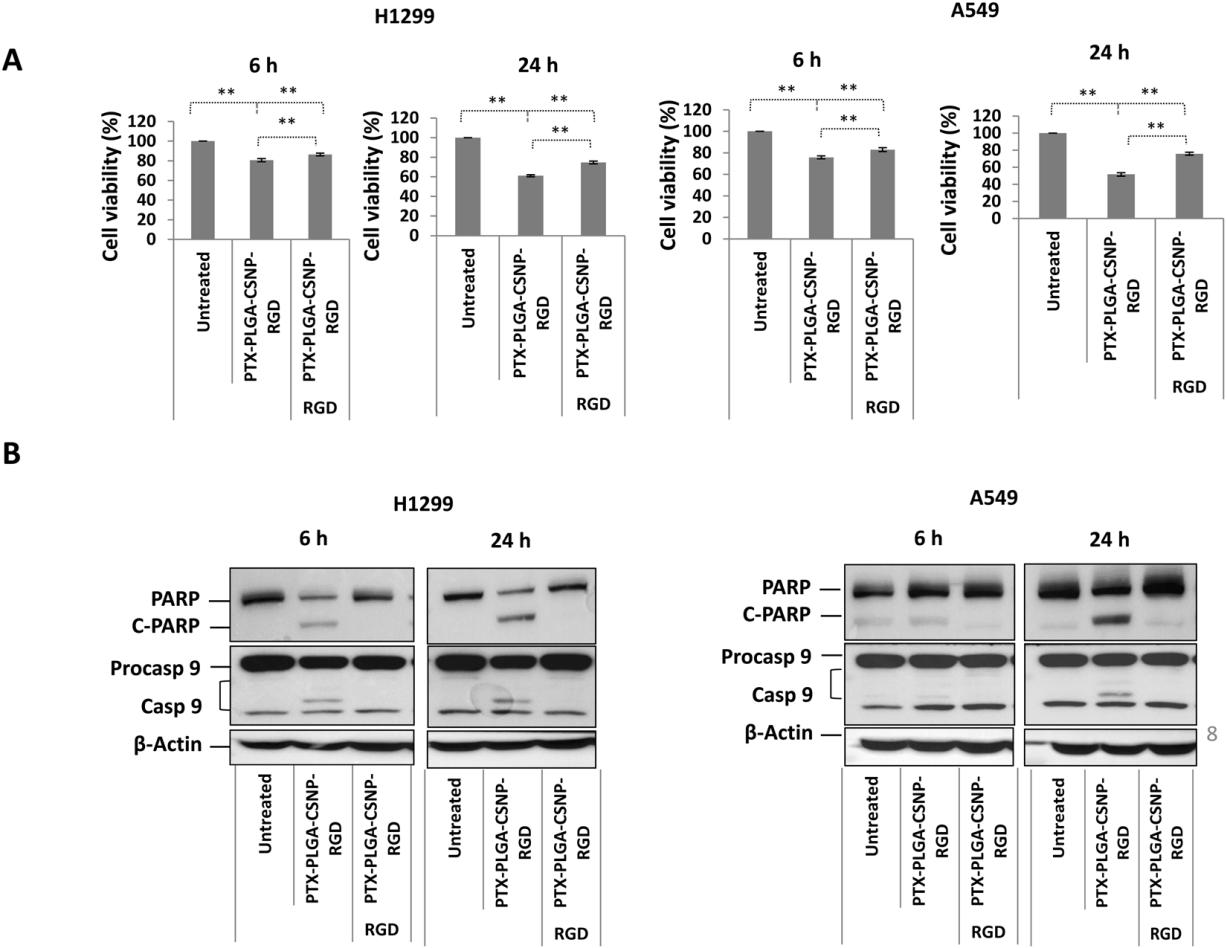
Integrin α_v_β_3_ receptor blocking study. (**A**) Cell viability and (**B**) western blot analysis of apoptotic proteins (PARP and Caspase 9) in nanoparticle-treated H1299, A549 cells. One test group (integrin blocking group) in each experiment was incubated with excess RGD peptide (10X) for 1 h prior to PTX-PLGA-CSNP-RGD treatment for 6 h and 24 h. The second test group received no free RGD incubation but received PTX-PLGA-CSNP-RGD treatment similar to the integrin blocking group. Untreated groups served as control group. Expression of PARP and caspase proteins were analyzed from different parts of the same gel; cut to separate the two time points and assembled as one figure for each cell line. (n = 3); *p < 0.05; **p < 0.01; NS, non-significant.

To understand whether the cellular uptake of targeted nanoparticles is facilitated through RGD-integrin α_v_β_3_ receptor interaction, we monitored the delivery efficiency in terms of PTX cytotoxicity and its effect on the levels of active caspase 9 (Casp 9) and Cleaved (C)-PARP in H1299, A549, and H1975 cells. We used IC_50_ equivalents of PTX for A549 (~12.5 nM), H1299 (~28.0 nM), and H1975 (~2.0 nM) cells. An excess of free RGD was initially used to block the integrin receptors prior to the treatment of cells with PTX-PLGA-CSNP-RGD. The effect of RGD blocking and the subsequent PTX-PLGA-CSNP-RGD treatment are shown in Figs 6 and S9. The cell viability of RGD pre-incubated groups showed low cytotoxicity due to poor cell uptake of PTX-PLGA-CSNP-RGD and, thereby, reduced PTX cellular accumulation. However, cells that did not receive free RGD peptide showed enhanced cytotoxicity, indicating that the uptake of PTX-PLGA-CSNP-RGD occurred *via* RGD-integrin α_v_β_3_ receptor interaction to cause prominent PTX toxicity, especially at 24 h. The observation was consistent in all three NSCLC cell lines studied. For instance, in H1975 cells the 6 h cell viability of the RGD pre-incubated group was 80.6%, in contrast to the cell viability of the no-RGD (71.3%; *p* < 0.01) group [Figure S9A]. At 24 h, RGD blocking was more effective in H1975 cells, which showed 75.4% viability in the RGD group versus 53.9% (*p* < 0.01) for the no-RGD group. This effect corresponded to the greatest integrin α_v_β_3_ expression levels among the studied cell lines.

Figures 6B and S9B show the western blot analysis of apoptotic markers (caspase 9 and PARP cleaved products) from the integrin α_v_β_3_ blocking study. The quantification values of C-PARP and caspase 9 bands were normalized to actin and represented as fold changes over untreated control (Table ST3). Notably, the induction of caspase 9 and C-PARP was partially halted by integrin α_v_β_3_ blocking, and may be attributed to poor cell uptake of PTX-PLGA-CSNP-RGD nanoparticles.

Here, the accumulation of PTX because of efficient cell uptake of PTX-PLGA-CSNP-RGD induced the cleavage of caspase 9 (45 kDa) to a shorter 35-kDa fragment. This process ultimately results in ordered activation of a group of caspases, leading to apoptosis^57^. In the present study, the enhanced cellular entry of PTX-PLGA-CSNP-RGD account for the accumulation of PTX and its cytotoxic activity, causing apoptosis. Caspase 9 is the initiator caspase that signals the triggering of apoptosis in cells undergoing therapeutic or environmental stress^58^. Though paclitaxel-induced apoptosis involves different types of caspases (3, 7, 8 or 9), the requirement for a caspase or no caspase is still arguable and may depend on the cell type and sensitivity of the cell lines to paclitaxel^57,59,60^. Here we looked at the caspase 9 activated product that marks the apoptosis event in PTX treated cells because of its prominent expression levels across the experiments. The caspase 9 activation observed was supported by the induction of PARP cleavage product in the PTX treated groups. PARP is involved in the DNA repair mechanism of cells under therapeutic stress^61^; in the present study, the inducing factor is PTX. PARP is a substrate for caspase 3, a marker of an apoptosis cascade event^61^. The presence of C-PARP product (85 kDa) was obvious in the no-RGD group, in contrast to the RGD –pre-incubated group. Quantification of C-PARP and activated caspase 9 products in A549, H1299, and H1975 cells indicated that the cleavage was significantly halted as a result of integrin α_v_β_3_ blocking, affecting cell uptake of PTX-PLGA-CSNP-RGD. Overall, the results indicate that the specificity of RGD-integrin α_v_β_3_ receptor interaction has a significant effect on RGD-modified nanoparticle cell uptake, and it successfully facilitated the cytotoxic activity of nanoparticle encapsulated-PTX.

### Differential toxicity of PTX-PLGA-CSNP-RGD in NSCLC cells and normal lung fibroblasts

We next compared the efficiency of targeted (PTX-PLGA-CSNP-RGD) and non-targeted (PTX-PLGA-CSNP) systems in terms of cell viability and cellular biochemical changes causing caspase 9 activation and PARP cleavage among NSCLC cell lines (H1299, A549, H1975) and normal lung fibroblasts (MRC-9). Figure S10 shows the cell viability and western blot data obtained for H1299, A549, H1975, and MRC-9 cells treated with PTX-PLGA-CSNP-RGD or PTX-PLGA-CSNP for 24 h and 48 h. PTX-PLGA-CSNP-RGD-treated H1299, A549, and H1975 cells were significantly less viable than PTX-PLGA-CSNP-treated cells at 24 h. The difference in cell viability between targeted and non-targeted PTX delivery groups was higher in H1975 cells. At 24 h, the average cell viability of the PTX-PLGA-CSNP-treated cells was 74.7%, whereas the viability of the PTX-PLGA-CSNP-RGD-treated cells was 55.1% (*p* < 0.01). At 48 h, this difference increased to 50.1% and 38.3% (*p* < 0.01), respectively, for PTX-PLGA-CSNP- and PTX-PLGA-CSNP-RGD-treated groups. However, the H1299 cells with low level integrin receptor expression showed a small difference in cell killing efficiency between targeted and non-targeted delivery groups, suggesting that the integrin receptor expression levels influence to the cell uptake and cytotoxicity of PTX-PLGA-CSNP-RGD.

Despite high expression of integrin receptors and enhanced cell uptake of PTX-PLGA-CSNP-RGD, MRC-9 cells were relatively less sensitive to PTX (28.0 nM) toxicity than were cancer cells. At both time points, the cell viability was not significantly different between cells treated with PTX-PLGA-CSNP and PTX-PLGA-CSNP-RGD. Compared with untreated controls, the toxicity was moderate, and not significant (*p* > 0.05).A general justification for low cytotoxic response of normal cells compared to cancer cells is the differences in their respective proliferation rates^62^. In addition, cancer cells often have diminished DNA damage repair capacity, affecting DNA replication, resulting in preferential cell death *via* apoptosis. Chemotherapeutic drugs act *via* mechanisms involving DNA damage, protein deterioration, microtubule aggregation, and/or mitochondrial damage^63^. Further, toxic response by cells also depends on the drug concentration reached in cellular compartments where cellular targets of respective drugs are located. Therefore, in addition to the targeted receptor expression levels, all the above-mentioned factors might have contributed to the differential toxicity observed between cancer cells and normal cells. Notably, empty nanoparticles (PLGA-CSNP-RGD) did not cause any significant toxicity in both cancer cells (A549) and in normal fibroblast cells (MRC-9) when incubated for 24 h and 48 h at concentrations ranging from 0–100 µg/mL [Figure S11].

Since the α_v_ subunit of integrin receptor (vitronectin receptor) is expressed in all cell lines studied, we measured the α_v_ expression levels in PTX-PLGA-CSNP-RGD- treated NSCLC cells. Western blot data showed that the levels of α_v_ were significantly reduced as a result of RGD targeting [Figure S10B and Table ST4]. This is a clear evidence of PLGA-CSNP-RGD interaction with integrin receptor. The endogenous levels of caspase 9 and C-PARP products in NSCLC cells reflected the findings from the cell viability studies. The C-PARP products were significantly enhanced in H1975 cells upon PTX-PLGA-CSNP-RGD treatment at 24 h and 48 h, compared with A549 and H1299 cells. Compared with cells receiving non-targeted PTX-PLGA-CSNP, the C-PARP levels were enhanced in cells receiving targeted treatment. No apparent C-PARP expression was observed in untreated controls. In the MRC-9 blots, a moderate decrease in α_v_ expression levels was observed, especially at 48 h, indicating integrin receptor blocking by PTX-PLGA-CSNP-RGD interaction. C-PARP expression was observed at 24 h and 48 h in both treatment groups, suggesting apoptosis. However, compared with stronger C-PARP product in NSCLC cells (H1299, A549 and H1975 cells) in PTX-PLGA-CSNP-RGD groups (*p* < 0.05), MRC-9 cells showed moderate-to-low C-PARP expression. Though caspase 9 expression was detected in MRC-9 cells the difference between PTX-PLGA-CSNP and PTX-PLGA-CSNP-RGD was not significant. A slightly elevated level of caspase 9 was seen in PTX-PLGA-CSNP-RGD group at 48 h compared untreated control and PTX-PLGA-CSNP. Hence, our results show that PTX-PLGA-CSNP-RGD induced differential toxicity in NSCLC and normal lung fibroblasts.

Cellular uptake and rapid release of drug is generally preferable for achieving a quick therapeutic response. However, slow and sustained delivery is often required for prolonged therapy of cancer. PLGA nanoparticles not only protect the drug from early release but also control the release in a slow and sustained fashion. Moreover, from the cell uptake study we observed that PTX uptake is significantly higher in targeted drug delivery compared to free PTX and non-targeted system. This pattern suggests that, at the time points of the study, PTX-PLGA-CSNP-RGD accumulation in cells is significant however therapeutic activity was comparable to free PTX. It is therefore we attributed that in our study the therapeutic efficiency was corresponding to the slow drug release pattern of PTX-PLGA-CSNP-RGD system. This observation suggests that targeted nanoparticles would induce therapeutic efficiency, but in a controlled and prolonged fashion which might be useful in treating tumors *in vivo*.

### Efficiency of PLGA-CSNP-RGD in targeted delivery of alternative drug CDDP

**Figure 7:**
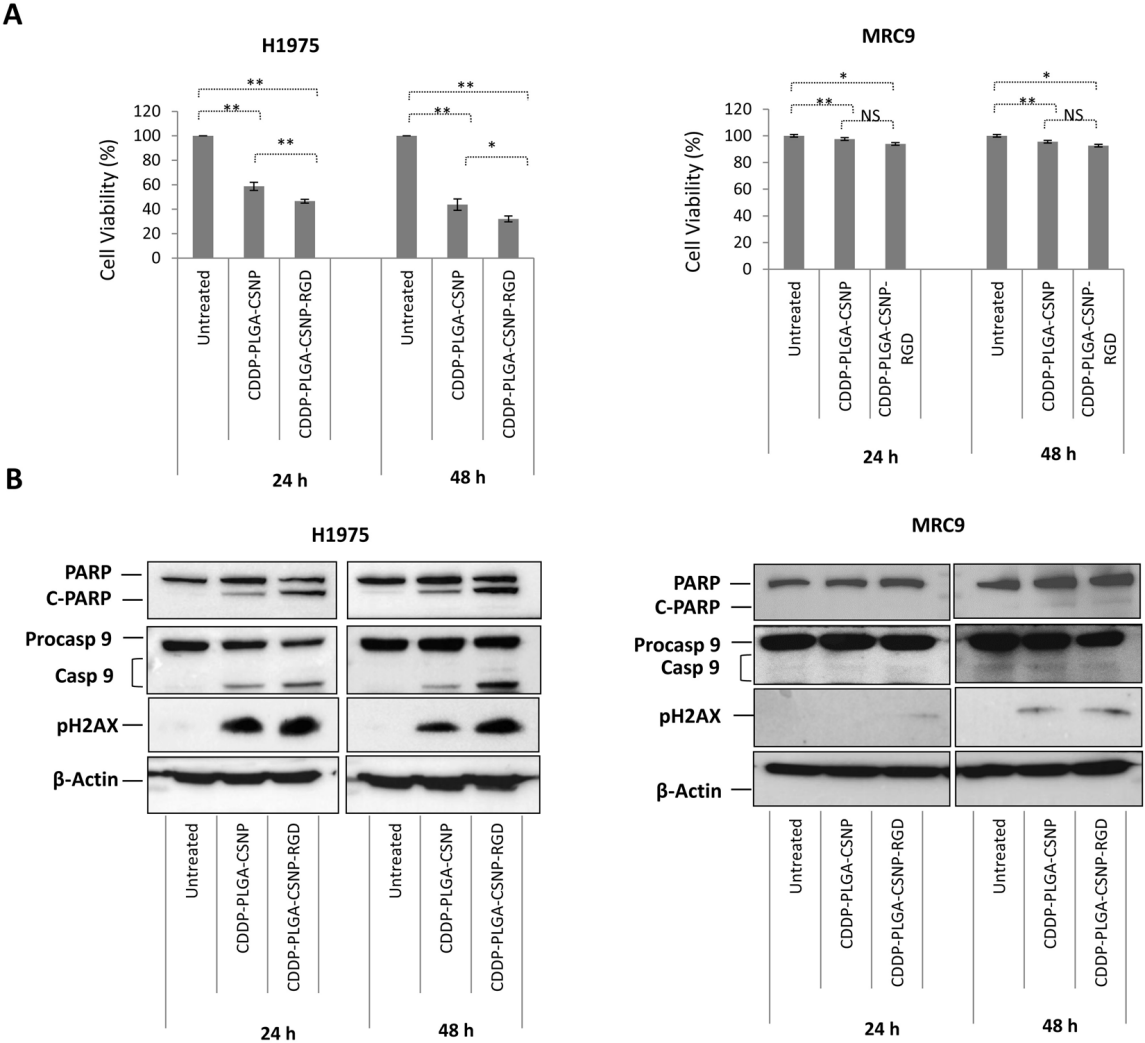
Efficiency of PLGA-CSNP-RGD in targeted delivery of alternative drug CDDP in lung cancer and fibroblast cells. (**A**) Cell viability, (**B**) western blot analysis in H1975 and MRC-9 cells. Cells were treated with CDDP-PLGA-CSNP (non-targeted) and CDDP-PLGA-CSNP-RGD (targeted) for 24 h and 48 h and compared with untreated controls. CDDP doses were 10 µM for H1975 and 40 µM for MRC-9. Proteins expression of PARP_,_ caspase 9 and H2AX were analyzed from different parts of the same gel, and cut to separate the two time points and assembled as one figure for each cell line. (n = 3); *p < 0.05; **p < 0.01; NS, non-significant.

It would be advantageous if the PLGA-CSNP-RGD system could be used to deliver therapeutics other than PTX. Due to its solubility in water, mechanism of action, and therapeutic potency, cisplatin (CDDP) is among the first line chemotherapeutic used for lung cancer therapy. Therefore, CDDP is also suitable model drug to verify the efficacy of the PLGA-CSNP-RGD carrier for lung cancer therapy. We prepared CDDP- PLGA-CSNP-RGD that has shown good physicochemical properties to be evaluated in NSCLC [Figures S12 and S13], lung fibroblasts and normal brochio-epithelial cells (late discussed in this manuscript). For cisplatin loaded nanoparticles (CDDP-PLGA-CSNP-RGD) the drug loading efficiency was 9.16% and encapsulation efficiency was 44%. We examined CDDP-PLGA-CSNP-RGD induced cell killing [Fig. 7A] and apoptotic protein levels [Fig. 7B], compared with non-targeted CDDP-PLGA-CSNP. In H1975 cells, CDDP-PLGA-CSNP-RGD treatment (10 µM CDDP equivalent) resulted in enhanced cell killing efficiency compared with CDDP-PLGA-CSNP at 24 h (*p* < 0.01) and 48 h (*p* < 0.05) time points respectively. However, MRC-9 cells treated with a CDDP-PLGA-CSNP-RGD (40 µM CDDP equivalent) resulted in negligible cell killing. No significant difference in cell killing was observed between targeted and non-targeted delivery of CDDP at either time point. This result was similar to the observations of cell viability with PTX-PLGA-CSNP-RGD or PTX-PLGA-CSNP treatment in MRC-9 cells.

In addition to the apoptotic protein markers, we also verified the expression of γH2AX in H1975 and MRC-9 cells in protein extracted from the cell viability study. CDDP induces DNA double-strand breaks (DSB), leading to an elevated level of γH2AX^64^. Phosphorylated H2AX (pH2AX) is considered a suitable biomarker to assess DSBs caused by chemotherapy^65^ or radiation^66^. Figure 7B and Table ST5 show the western blot analysis and fold changes in apoptotic proteins of H1975 and MRC-9 cells treated with CDDP-PLGA-CSNP-RGD or CDDP-PLGA-CSNP for 24 h and 48 h, respectively. Blots of H1975 cells show enhanced C-PARP and caspase 9 in CDDP-PLGA-CSNP-RGD-treated cells compared with CDDP-PLGA-CSNP-treated cells (*p* < 0.01) and untreated controls (*p* < 0.01) at 24 h. However, the difference between non-targeted and targeted CDDP delivery groups was not clear (*p* > 0.05). However, at 48 h H1975 cells showed a significant γH2AX expression in CDDP-PLGA-CSNP-RGD treated groups compared to non-targeted and untreated controls. At 48 h, caspase 9 expression also showed a similar trend in CDDP-PLGA-CSNP-RGD-treated groups, but not in C-PARP expression. Similar trends in cell viability and in western analysis of apoptotic protein levels were observed in H1299 and A549 cells treated with CDDP-PLGA-CSNP-RGD [Figure S14]. In contrast, western blot analysis of MRC-9 proteins showed negligible induction of C-PARP (89 kDa); the levels of casapse 9 (37 and 35 kDa) were undetectable [Fig. 7B]. This result is a clear indication of the low toxicity response of MRC-9 cells to CDDP-PLGA-CSNP-(RGD+/−). This finding also suggests that high integrin α_v_β_3_ expression levels in MRC-9 cells have no or little influence on the cytotoxic response to RGD-based targeted nanoparticle delivery of chemotherapeutic drugs.

### Integrin α_v_β_3_ negative NHBE cells showed negligible cytotoxic response to PTX- or CDDP-PLGA-CSNP-RGD

In the next step, we used normal human bronchial epithelial cells (NHBE) to evaluate the efficiency of PTX- or CDDP- PLGA-CSNP-RGD in inducing cytotoxicity. Integrin α_v_β_3_ expression in NHBE cells is negligible, and as per our hypothesis there would be no significant difference in cell uptake of PLGA-CSNP-RGD- or PLGA-CSNP-carrying chemotherapeutics. Moreover, NHBE cells are known to be less sensitive to anti-cancer drugs than are neoplastic cells^67,68^. Here we carried out cell uptake, cell viability, and apoptotic protein expression studies in NHBE cells treated with PLGA-CSNP-RGD or PLGA-CSNP carrying FluTax, PTX, or CDDP.

**Figure 8:**
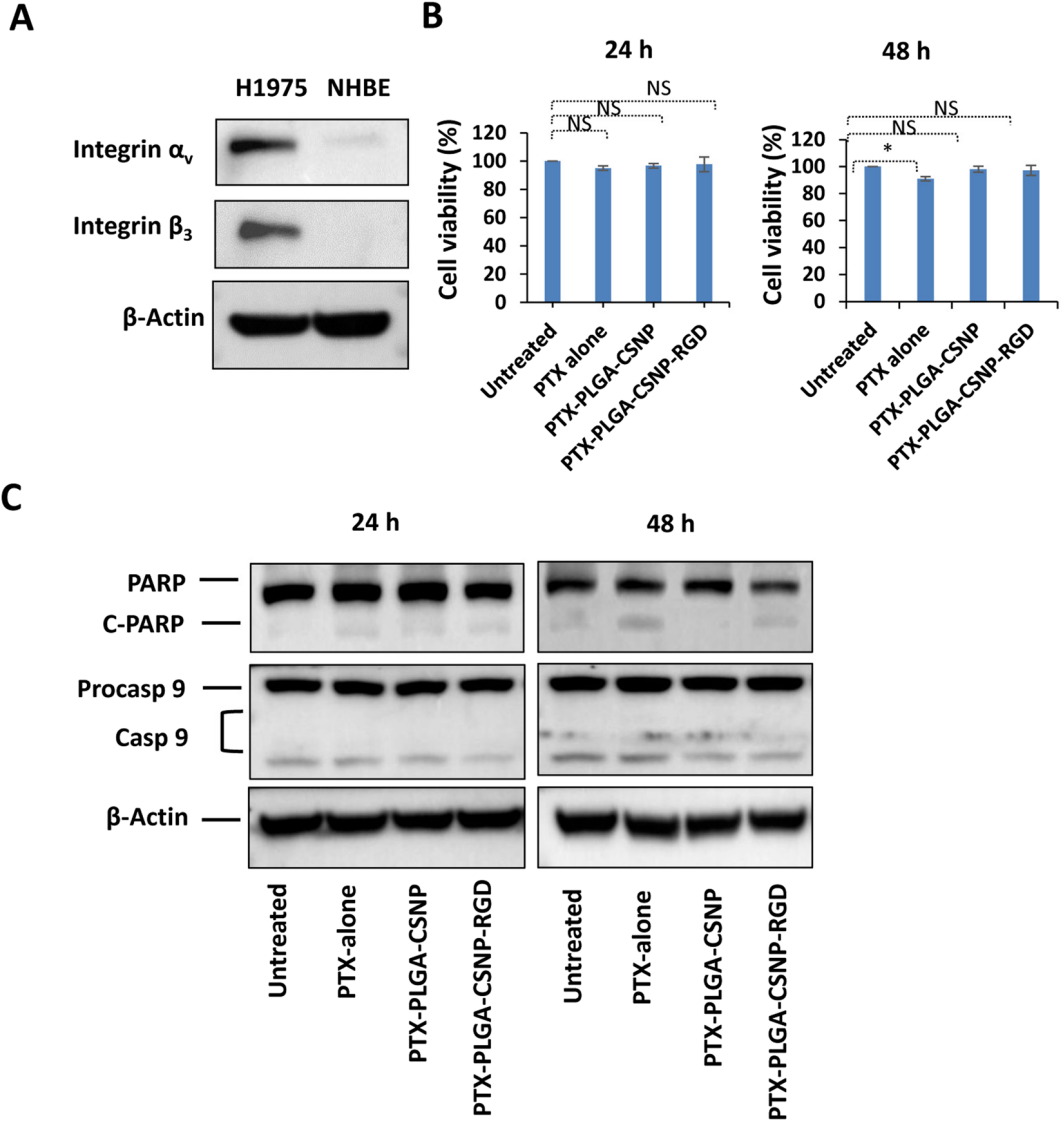
PTX-PLGA-CSNP-RGD treatment effect in NHBE cells. (**A**) Baseline expression of integrin α_v_β_3_ in NHBE cells versus H1975 cells. Proteins expression of integrin α_v_ and β_3_ were analyzed in two separate gels and assembled in one figure. (**B**) Cell viability and (**C**) western blots for apoptotic proteins (PARP and caspase 9). Cells were treated with free PTX, PTX-PLGA-CSNP, PTX-PLGA-CSNP-RGD in 28.0 nM PTX equivalents. Proteins expression of PARP, caspase 9 and actin was analyzed from different parts of the same gel for each time-point and protein analysis for each time point was performed on a separate gel. NS, non-significant.

Figure 8A shows the western blot data of integrin α_v_β_3_ expression levels in NHBE compared with H1975 NSCLC cells, indicating integrin α_v_β_3_ in NHBE is negligible. Further, cell uptake data show no significant difference between FluTax-PLGA-CSNP-RGD or FluTax-PLGA-CSNP at 6 h and 24 h of treatment [Figure S7A and B (*p* > 0.05)]. Cell viability assays with PTX- or CDDP- PLGA-CSNP-RGD in NHBE cells showed no significant difference, compared with their respective non-targeted counterparts [Figs 8B and S14]. PTX -PLGA-CSNP-RGD or CDDP -PLGA-CSNP-RGD treatment in NHBE cells produced minimal cell death [Figs 8B and S14]. Further, western blot data showed that PTX-PLGA-CSNP-RGD, CDDP-PLGA-CSNP-RGD, and their non-targeted counterparts did not trigger significant expression of apoptotic proteins [Figs 8C and S14, Table ST6]. C-PARP was less expressed when treated with free PTX or PTX-PLGA-CSNP-RGD, and were not significantly higher than non-targeted PTX-PLGA-CSNP. This low cytotoxicity of PTX in normal cells was explained by a previous report stating that 10 nM to 50 nM PTX was not cytotoxic, while cancer cells showed significant sensitivity and cell death when treated with these doses^69^. It is important to note that in the present study, the PTX concentration in PTX-PLGA-CSNP-RGD treatment was enough to kill more than 50% of cancer cells, which may be well below the cytotoxic concentration of PTX in NHBE cells. A similar trend was observed with CDDP or CDDP-PLGA-CSNP-(RGD+/−) treatments for either 24 h or 48 h. This lack of significant cytotoxicity in normal cells supports the use of an integrin α_v_β_3_-targeted RGD-modified PLGA-CSNP drug delivery system towards lung cancer cells.

## Conclusion

In the present study, we developed a targeted nanoparticle system based on PLGA and chitosan, modified with linear RGD peptide for integrin α_v_β_3_-targeted delivery of anti-cancer drugs to lung cancer cells. Among a panel of cell lines we evaluated for integrin expression levels and H1975, A549 and H1299 cells with higher α_v_β_3_ levels were selected for our studies. Further, MRC9 cell line was selected to represent the lung fibroblasts, with transient overexpression of integrin α_v_β_3_ receptors. In addition, integrin negative NHBE cell line was also included in our study. Studies in these cell lines with different integrin expression levels give better insights into integrin targeted nanoparticle based chemotherapeutic delivery, their efficacy and toxicity. PLGA-CSNP-RGD nanoparticles showed selective delivery of PTX to integrin α_v_β_3_-overexpressing cells, leading to significant toxicity compared with its non-targeted counterpart. The PTX- PLGA-CSNP-RGD particles induced apoptosis *via* G2/M cell cycle arrest and by activating caspase 9 and PARP cleavage. In normal lung fibroblasts expressing integrin α_v_β_3_ receptors, the cell uptake of PLGA-CSNP-RGD was comparable to that of cancer cells; however, the low toxicity from chemotherapeutic drugs might be attributed to their poor sensitivity. Normal broncho-epithelial cells with negligible integrin α_v_β_3_ expression experienced no apparent toxicity with any of the nanoformulations, indicating that PTX- or CDDP- PLGA-CSNP-RGD has no effect in normal cells that do not express their target receptors.

The present study also demonstrated that PLGA-CSNP-RGD is a potential delivery system for cisplatin, which had a similar cytotoxic effect in lung cancer cells, but spared lung fibroblasts and normal cells. In addition, our studies underline that other than the highly explored anti-angiogenic therapy, the potential of RGD peptide-integrin α_*v*_β_3_ receptor interaction can also be harnessed for cell-specific delivery of therapeutics using nanoparticles, especially in integrin overexpressing lung cancer cells. However, the notion that normal cells always express integrin α_v_β_3_ receptors at very low levels compared to cancer cells may not be true, since transient overexpression of such receptors are noticed in many normal cells or healthy fibroblasts that surround the tumor. Therefore, our study suggests that judicious evaluation of different normal cell lines is required while studying the efficacy of a targeted nanoparticle drug delivery system for cancer therapy. This study thus provides an important insight into integrin (α_v_β_3_) receptor targeted nanoparticle-drug delivery towards lung cancer cells and its effect in normal cells showing variable expression levels of integrin (α_v_β_3_) receptor-expression. Taken together, PLGA-CSNP-RGD could be a potential tool for the preferential delivery of chemotherapeutic drugs to lung cancer cells.

## Materials and Methods

### Chemicals

Acid-terminated Poly-l-lactate-co-glycolic acid (PLGA, MW 25000; lactide:glycolide 50:50, chitosan (50–190 kDa; 75–85% DDA), Cis-diamine platinum (CDDP), O-phenylenediamine, poly-vinyl alcohol (PVA; MW 89KDa-98KDa, 99+% hydrolyzed), and RGD peptide (GRGDSP) were purchased from Sigma-Aldrich, USA. NHS-PEG_12_-Maliemide and 2-iminothiolane were obtained from Thermo-Fischer Scientific, Rockford, IL, USA. Paclitaxel and paclitaxel-oregon green 488 (FluTax) were procured from Molecular Probes, Eugene, OR, USA.

### Cell lines and cell culture

A549, H1299, H1975, and MRC-9 cells were purchased from American Type Culture Collection, USA. The lung cancer cells were grown and maintained in RPMI-1640 media whereas normal lung fibroblast cells (MRC-9) were maintained in MEM media. The complete media contained 10% FBS (Corning, Manassas, VA, USA), and 1% antibiotic and antimycotic solution (Sigma-Aldrich, USA). NHBE cells and its specific growth medium (BEGM Bulletkit) were obtained from Lonza, Walkerswille, MD, USA. The cells were grown under the conditions specified by the manufacturers.

### Synthesis of RGD peptide-conjugated chitosan

RGD-Chitosan (RGD-CS) was synthesized by coupling GRGDSP peptide to chitosan amino groups *via* maleimide-PEG-NHS cross-linker. Briefly, the first step was to introduce a sulfhydryl group into RGD peptide (5.5 mM) by reacting it with 2-iminothiolane (16.5 mM) at RT under mild stirring for 4 h in Na_2_HPO_4_: EDTA buffer (pH 8.0). Thiolated-RGD peptide was then allowed to react with maleimide group of maleimide-PEG-NHS linker (22 mM) in pH 7.2 Na_2_HPO_4_: EDTA (0.2 M) buffer and was kept at RT under mild shaking overnight. The resulting product, RGD-PEG-NHS, was then allowed to react with chitosan. Chitosan (0.2%) was initially solubilized in pH 5.5 acetate buffer (0.2 M) and was then equilibrated in double-distilled water (DDH_2_O), followed by pH 7.2 Na_2_HPO_4_: EDTA buffer (0.2 M). RGD-PEG-NHS (2 mM) was added to this CS solution (5 mL) and the mixture was kept under mild shaking for 2 h at RT and was dialyzed overnight against DDH_2_0 in 10 kDa cut-off nitrocellulose tubing. The resultant product was CS-PEG-RGD, which was labelled CS-RGD and stored at 4 °C until use. The amount of RGD conjugated to CS was measured by the standard Bicinchoninic acid (BCA) assay (Thermo Fischer Scientific, USA) for proteins.

### Preparation and characterization of targeted PLGA nanoparticles loaded with chemotherapeutics

PLGA nanoparticles were prepared using emulsification solvent evaporation^70^ or double emulsion method^71^ for the delivery of PTX or CDDP respectively. Briefly, 20 mg/mL of PLGA and 2 mg PTX was dissolved in 1 mL chloroform and was mixed with 2% PVA solution (4 ml) and emulsified under ultra-sonication (20 KHz, 30% amplitude) for 1 minute in ice. For CDDP-PLGA nanoparticles CDDP was added to the 2% PVA solution (1 mg/ml) and emulsified in chloroform (4 mL) to form a primary emulsion. The primary emulsion was then added to an excess of 2% PVA (80 mL) and sonicated for 4 min in 30-second pulses to form a secondary emulsion. Both the PTX and CDDP containing emulsions were then stirred in a fume hood overnight at RT to allow evaporation of chloroform and hardening of the nanoparticles. The nanoparticles were then purified by 3x centrifugation at 15000 rpm for 20 minutes at 4 °C. The purified nanoparticles were stored at 4 °C in nanopure water. The drug-loaded PLGA nanoparticles were then coated with chitosan or RGD-chitosan. Two mL of chitosan or RGD-chitosan solution (0.2%) was taken in a glass tube and stirred using a magnetic stirrer at 300 rpm for 2 h at RT. Then, 1 mg/mL of PTX-PLGA nanoparticles was added dropwise with a micropipette, under stirring conditions. After 2 h, unreacted CS or CS-RGD was removed using centrifugation at 15000 rpm for 20 minutes at 4 °C. The resulting pellet was resuspended in nanopore water and filter centrifuged using Amicon centrifugal filters (10 KDa MWCO). The concentrated PTX-PLGA-CSNP-(+/−RGD) was resuspended in nanopure water and stored at 4 °C until use.

### Physicochemical characterization of nanoparticles

Particle size, zeta potential, and polydispersity index of the nanoparticles were measured using a Brookhaven, ZetaPALS instrument. Samples were diluted to 1:10 of the original concentration in nanopure water in disposable cuvettes; we conducted 10 individual runs in triplicate. Transmission electron microscopy (TEM) images of the particles were obtained *via* Hitachi-H7600 microscope at the Oklahoma Medical Research Foundation core imaging facility. Particles were deposited in copper grids and stained using 2% phosphotungstic acid before imaging. For estimating the drug loading (%)the nanoparticles were freeze-dried for 12 h at −50 °C at 0.006 mbar pressure (Labconco, Kansas City, MO). HPLC (for PTX) or o-phenylenediamine (OPDA) spectrophotometric reading (for CDDP) was used for estimation of the drug concentration in nanoparticles. The amount of drug in the supernatant after the purification step was also measured by respective techniques for PTX and CDDP to calculate the encapsulation efficiency. To carry out HPLC measurements, PTX-PLGA-CSNP-RGD samples were digested in HPLC-grade acetonitrile under sonication for 2 min at 20 MHz and 30% amplitude. The samples were then kept in a fume hood in the dark for 24 h to extract the loaded drug. Then, the samples were centrifuged at 15000 rpm for 20 min. The supernatant was filtered twice using 200-nm-pore PTFE syringe filters and analyzed for PTX content using HPLC analysis. The HPLC system (Beckman System Gold HPLC) was equipped with a Beckman Model 126 pump and an Applied Biosystems 783 A programmable absorbance detector. HPLC solvents consisted of water containing 0.1% trifluoroacetic acid (solvent A) and acetonitrile containing 0.1% trifluoroacetic acid (solvent B). A Sonoma C18 (ES Industries, 10 µm, 100 Å, 4.6 × 250 mm) column was used with a flow rate of 1.5 mL/min. The HPLC gradient system began with an initial solvent composition of 80% A and 20% B for 2 minutes, followed by a linear gradient to 100% B in 15 minutes, after which the column was re-equilibrated. The absorption detector for PTX was set at 227 nm. An SRI Instruments Model 302 six-channel USB chromatography data system was used for data acquisition, operating on PeakSimple software. For calibration curves, 50 µl of paclitaxel standard with concentrations of 5, 10, 25, 50, and 250 µg/ml in AcCN was injected into HPLC. Calibration curves were constructed using peak area of the analyte concentrations. The linearity was evaluated in the concentration range of 5–250 µg/ml of paclitaxel with regression coefficient (R2) of 0.9999, slope of 75.12, and intercept of 30.961. The precision of this method was between 1.9 and 5.7%CV at the above concentrations. Three replicates were determined at each concentration. The sensitivity (lower limit of detection) is 0.25 µg/ml (S/N = 3). The accuracy is 105.4 ± 9.2. For accuracy, three paclitaxel samples of 50 µg/ml concentration made on different days were used.

CDDP content in the nanoparticles was determined by *o*-phenylenediamine (OPDA) -based spectrophotometric assay^72^. Briefly, a known quantity of CDDP-PLGA-CSNP-RGD particles was digested in dimethyl formamide (DMF) by sonication for 2 min at 20 MHz and 30% amplitude, and was extracted overnight in the dark. The supernatant was mixed with OPDA solution (1.4 mg/mL) in DMF and was heated at 90 °C for 30 min to form a colored complex. The absorbance maximum of this complex was 706 nm. The unknown sample concentration was obtained from a CDDP-OPDA standard graph (*R* ^2^ = 0.9996). The drug encapsulation and loading efficiencies were calculated using the following equation^38^; Drug loading % = (Drug content in nanoparticles/total weight of the drug loaded nanopartices) × 100. Encapsulation efficiency % = [(weight of the drug used in the formulation-weight of the drug lost in the medium)/weight of the drug used in the formulation] × 100.

### *In vitro* drug release

*In vitro* drug release was carried out by dialyzing a known concentration of drug-loaded nanoparticle solution against PBS (pH 7.4) using Slide-A-Lyzer MINI dialysis devices (Thermo Fisher Scientific, USA) of 3.5 K cutoff pore size. Briefly, 500 µL of PLGA-CSNP-RGD (which contained 3.23 µg of PTX based on drug loading) solution was pipetted into a Slide-A-Lyzer MINI dialysis device. The device was then inserted into 1.3 mL of PBS (pH 7.4) containing microfuge tubes and kept at 37 °C under shaking (150 rpm). At predetermined intervals, each tube was collected, the nanoparticle solution in Slide-A-Lyzer MINI dialysis device was recovered, centrifuged and the pellet was collected. The pellet was then digested in HPLC grade acetonitrile overnight to isolate the retained drug and the concentration was measured using HPLC (for PTX- PLGA-CSNP-RGD). The retained drug quantity was subtracted from the initial drug quantity in the nanoparticle added to dialysis device and back-calculated the amount of drug released in the medium.

For CDDP-PLGA-CSNP-RGD, the drug concentration in the dialysis medium recovered from the microfuge tube was measured by OPDA spectrophotometric method for drug release percentage calculation. The drug release was followed until 96 h.

### Studies in cell lines

#### Optimization of RGD concentration

RGD concentration in PLGA-CSNP was optimized to achieve the best possible nanoparticle-cancer cell interaction mediated by integrin α_v_β_3_ receptor expression. First, PLGA nanoparticles were loaded with fluorescent dye Rhodamine B (RB; 0.1 mg/mL in 2% PVA) using the double emulsion technique, as described earlier. The RB-loaded PLGA nanoparticles were purified by 3X centrifugation at 15000 rpm for 20 min in 4 °C. In five separate glass tubes, 1 mL of 0.2% CS/RGD-CS mixture in 1:0, 1:2, 1:4, 1:8, and 1:16 ratios were added, respectively. About 1 mg/mL of RB-PLGA nanoparticles were added drop-wise into these RGD-chitosan solutions under stirring (300 rpm) in RT for 2 h to prepare RGD-CS-coated, RB-carrying nanoparticles. The resulting nanoparticle suspensions were purified by centrifugation at 15000 rpm for 20 min at 4 °C and concentrated by Amicon centrifuge filtration (3.5 K cutoff pore size). The RGD concentrations in each of these RB-PLGA-CSNP-RGD formulations were obtained by BCA assay and added to cells in culture in final RGD concentrations of 0, 0.085, 0.17, 0.34, or 0.68 µM.

Cell culture experiments were conducted to optimize RGD concentration in nanoparticles. Briefly, 1 × 10^5^ A549 cells per coverslip or 1.25 × 10^5^ A549 cells in each well of 6-well plates were seeded in complete RPMI-1640 medium. After 24 h, the medium was replaced with RB-PLGA-CSNP-RGD containing serum-free medium, and the cells were incubated for 6 h. Then, the medium was replaced with 10% serum-containing medium and was incubated until the 24 h time point. Cells in coverslips were fixed in 4% paraformaldehyde and nuclear-stained with DAPI for examination using a Nikon Ti (Japan) fluorescence microscope at 6 h and 24 h. Another batch of cells was harvested, counted, and placed in a 96-well black *µ*Clear –plate and read on an Envision microplate reader (Perkin Elmer) using a Cy5 filter (λ_625_ emission) for RB fluorescence. Background fluorescence was omitted and the values were calculated and graphically represented. The RB-PLGA-CSNP-RGD test group with the highest fluorescent intensity obtained that correlated with microscopic examination was identified as the optimal RGD concentration for PLGA-CSNP-based integrin α_v_β_3_-targeted drug delivery.

#### Cell uptake study

Briefly, A549 (1.25 × 10^5^ cells/well), H1299 (1.5 × 10^5^ cells/well), H1975 (1.5 × 10^5^ cells/well), and MRC-9 (2 × 10^5^ cells/well) cells were seeded in 6-well plates. After 24 h, the 10% serum- containing medium was replaced with serum-free medium containing fluorescent paclitaxel (FluTax® Oregon 488) formulations. FluTax was used in equivalent concentrations (2.0 nM) as free drug, and in RGD modified and unmodified nanoparticles to incubate with all the cell lines. The cell uptake of Free FluTax, FluTax-PLGA-CSNP-RGD,and FluTax-PLGA-CSNP were analyzed for fluorescence intensity of FluTax accumulated in cells using Envision plate reader at 6 h and 24 h post-incubation. Untreated cells were used as controls. In a parallel experiment, cells grown in coverslips were treated with FluTax-PLGA-CSNP-RGD and visualized under a fluorescence microscope for green fluorescence of FluTax using a blue excitation fluorescence filter.

### RGD competition assay: Integrin α_v_β_3_ receptor blocking studies

Cells were seeded in 6-well plates in complete RPMI-1640 medium. After 24 h, cells were incubated for 1 h, with or without 10 times molar excess (3.8 µM) of RGD peptide. The medium was then replaced with serum-free medium containing PTX-PLGA-CSNP-RGD loaded with a PTX dose of 12.5 nM (A549 and H1299 cells) or 2.0 nM (H1975). After 6 h, the medium was again replaced with 10% serum-containing medium and cells were incubated until 24 h. Untreated cells were used as controls. The cells were harvested at 6 h and 24 h, and cell viability was measured using the standard Trypan blue exclusion method^73^ (Ramesh, *et al*., 2003). Proteins were extracted in radio immunoprecipitation assay (RIPA) buffer (Sigma-Aldrich, USA) containing protease and phosphatase inhibitors (Roche, IN, USA) for western blot analysis.

### Cell viability

Cell viability assay was carried out using the standard Trypan blue exclusion assay^73^. Briefly, A549 (1.25 × 10^5^ cells/well), H1299 (1.0 × 10^5^ cells/well), H1975 (1.5 × 10^5^ cells/well), MRC-9 (2 × 10^5^ cells/well), and NHBE (2 × 10^5^ cells/well) cells were seeded in 6-well plates and treated with PTX-PLGA-CSNP-RGD or PTX-PLGA-CSNP with PTX doses of 12.5 nM (A549, H1299, MRC-9, NHBE) or 2.0 nM (H1975). After 24 h and 48 h, cells were harvested and stained with Trypan blue dye, and viable cells were counted.

### Apoptosis analysis

Apoptosis induction by PTX-PLGA-CSNP-RGD or PTX-PLGA-CSNP in A549 and H1299 cells was measured with the Alexa Fluor-488-Annexin-V/Propidium Iodide (PI) dual staining kit per the manufacturer’s instructions (Thermo Fisher Scientific, Eugene, OR. Briefly, cells were seeded in 6-well plates and treated with PTX-PLGA-CSNP-RGD or PTX-PLGA-CSNP for 24 h and 48 h, as described in the cell viability assay section. Free-PTX-treated and untreated cells served as controls. The harvested cells were washed in PBS, resuspended in 1X Annexin-V binding buffer, and incubated with 5 µl Alexa Fluor-488-Annexin-V, followed by 1 µl PI (1 mg/mL), and were then incubated for 15 min at RT. The cells were then subjected to flow cytometry. The data were plotted using -CellQuest^TM^ software. The percentage of apoptotic cells were presented as the sum of Q2 (Annexin-V-positive) and Q3 (Annexin-V/ PI-positive) quadrants^74^.

### Cell cycle analysis

After treatment of A549 cells (PTX 12.5 nM equivalent) and H1299 cells (PTX 28.0 nM equivalent) with PTX-PLGA-CSNP-RGD or PTX-PLGA-CSNP as described in the previous sections, cells were harvested at 24 h and 48 h and washed twice with ice-cold PBS (pH 7.4). Cells were fixed in 70% ethanol at 4 °C for 1 hour. After washing three times with ice-cold PBS (pH 7.4), the pellet was resuspended in 5 mL PI staining solution (0.5 mL of 1% sodium citrate, 50 µl of 10% Triton × 100, 250 µl of 1 mg/mL PI, 250 µl of 1 mg/mL RNase, and 3.950 µl of ice-cold nanopure water). Flow cytometry for cell cycle analysis was carried out after 10 min incubation at 37 °C^75^.

### Western blot analysis

Western blot analyses were performed according to previously reported procedures^6,9^. Briefly, protein extracts from cells treated with PTX- or CDDP- PLGA-CSNP-(RGD+/−) formulations and from control groups were run in sodium dodecyl sulfate-polyacrylamide gels and transferred onto Immobilon PVDF membrane (Millipore, Billerica, MA, USA). The membranes were then blocked using 5% milk in TBST buffer (pH 7.5), followed by blocking with primary antibodies (Cell Signaling Technology) in 1:1000 dilution in 5% milk in TBST buffer (pH 7.5) overnight at 4 °C. The next day, the membranes were washed and blocked with secondary antibodies (rabbit or mouse, depending on the primary antibodies; Sigma-Aldrich, USA) and imaged using Syngene GBOX after incubation with Clarity Western enhanced chemi-luminescence substrate (Biorad, USA). Beta actin was used as the internal control for all western blot experiments. Protein bands from western images were quantified using GelQuant.NET software.

### Statistical Analysis

Statistical analyses of all the experiments were performed using SAS software. A *P* value of < 0.05 was considered statistically significant. All experiments were repeated three times. The error bars represent the mean values with standard deviation (*SD*), unless otherwise indicated.

## Electronic supplementary material

Supplementary Data

## References

[1] Ozkok A, Edelstein CL (2014). Pathophysiology of Cisplatin-Induced Acute Kidney Injury. BioMed Res Int..

[2] Volkova M, Russell R (2011). Anthracycline Cardiotoxicity: Prevalence, Pathogenesis and Treatment. Curr Cardiol Rev..

[3] Ferrarotto R, Schetino G, Freitas D, Capelozzi V, Hoff PM (2010). Paclitaxel induced chronic fibrosing interstitial pneumonitis: a case report and review of the literature. Oncol Rev..

[4] Charpidou AG (2009). Therapy-induced Toxicity of the Lungs: An Overview. Anticancer Res..

[5] Aravind A (2012). Aptamer-labeled PLGA nanoparticles for targeting cancer cells. Cancer Nanotechnol..

[6] Amreddy N (2015). Tumor-targeted and pH-controlled delivery of doxorubicin using gold nanorods for lung cancer therapy. Int J Nanomedicine..

[7] Lu RM (2013). Targeted drug delivery systems mediated by a novel Peptide in breast cancer therapy and imaging. PLoS One..

[8] Muralidharan R (2016). Folate receptor-targeted nanoparticle delivery of HuR-RNAi suppresses lung cancer cell proliferation and migration. J Nanobiotechnology..

[9] Yang Y, Zhang YM, Chen Y, Chen JT, Liu Y (2016). Polysaccharide-based Noncovalent Assembly for Targeted Delivery of Taxol. Sci Rep..

[10] Guo Y, Wang L, Peng LV, Zhang P (2015). Transferrin-conjugated doxorubicin-loaded lipid-coated nanoparticles for the targeting and therapy of lung cancer. Oncol Lett..

[11] Shan D (2015). RGD-conjugated solid lipid nanoparticles inhibit adhesion and invasion of αvβ3 integrin-overexpressing breast cancer cells. Drug Deliv Transl Res..

[12] Danhier F, Breton AL, Préat V (2012). RGD-Based Strategies To Target Alpha(v) Beta(3) Integrin in Cancer Therapy and Diagnosis. Mol. Pharm..

[13] Liu Z, Wang F, Chen X (2008). Integrin alpha(v)beta(3)-Targeted Cancer Therapy. Drug Dev Res..

[14] Shukla R (2005). Tumor angiogenic vasculature targeting with PAMAM dendrimer–RGD conjugates. Chem. Commun..

[15] Teesalu T, Sugahara KN, Ruoslahti E (2013). Tumor-Penetrating Peptides. Front Oncol..

[16] Schiffelers RM (2003). Anti-tumor efficacy of tumor vasculature-targeted liposomal doxorubicin. J Control Release..

[17] Gandioso A, Cano M, Massaguer A, Marchán V (2016). A Green Light-Triggerable RGD Peptide for Photocontrolled Targeted Drug Delivery: Synthesis and Photolysis Studies. J. Org. Chem..

[18] Arosio D, Manzoni L, Araldi EM, Scolastico C (2011). Cyclic RGD Functionalized Gold Nanoparticles for Tumor Targeting. Bioconjugate Chem..

[19] Qu X (2016). Micro-CT Imaging of RGD-Conjugated Gold Nanorods Targeting Tumor *In Vivo*. J Nanomater..

[20] Damjanovich L, Albelda SM, Mette SA, Buck CA (1992). Distribution of integrin cell adhesion receptors in normal and malignant lung tissue. Am J Respir Cell Mol Biol..

[21] Singh B, Fu C, Bhattacharya J (2000). Vascular expression of the alpha(v)beta(3)-integrin in lung and other organs. Am J Physiol Lung Cell Mol Physiol..

[22] Zhang X (2016). Tumor targeting strategies for chitosan-based nanoparticles. Colloids Surf B Biointerfaces..

[23] Wang Y, Li P, Kong L (2013). Chitosan-Modified PLGA Nanoparticles with Versatile Surface for Improved Drug Delivery. AAPS PharmSciTech..

[24] Manca ML, Mourtas S, Dracopoulos V, Fadda AM, Antimisiaris SG (2008). PLGA, chitosan or chitosan-coated PLGA microparticles for alveolar delivery?: A comparative study of particle stability during nebulization. Colloids Surf B Biointerfaces..

[25] Mufti RE (2015). Implications of αvβ3 Integrin Signaling in the Regulation of Ca2+ Waves and Myogenic Tone in Cerebral Arteries. Arterioscler Thromb Vasc Biol..

[26] Kokkoli E, Ochsenhirt SE, Tirrell M (2004). Collective and single-molecule interactions of alpha5beta1 integrins. Langmuir..

[27] Hautanen A, Gailit J, Mann DM, Ruoslahti E (1989). Effects of modifications of the RGD sequence and its context on recognition by the fibronectin receptor. J Biol Chem..

[28] De la Fuente JM, Berry CC, Riehle MO, Curtis ASG (2006). Nanoparticle Targeting at Cells. Langmuir..

[29] Qian J (2016). Transarterial administration of integrin inhibitor loaded nanoparticles combined with transarterial chemoembolization for treating hepatocellular carcinoma in a rat model. World J Gastroenterol..

[30] Garg A, Kokkoli E (2011). pH-Sensitive PEGylated liposomes functionalized with a fibronectin-mimetic peptide show enhanced intracellular delivery to colon cancer cell. Curr Pharm Biotechnol..

[31] Garg A, Tisdale AW, Haidari E, Kokkoli E (2009). Targeting colon cancer cells using PEGylated liposomes modified with a fibronectin-mimetic peptide. Int J Pharm..

[32] Demirgöz D, Garg A, Kokkoli E (2008). PR_b-targetedPEGylated liposomes for prostate cancer therapy. Langmuir..

[33] Sharma N, Madan P, Lin S (2016). Effect of process and formulation variables on the preparation of parenteral paclitaxel-loaded biodegradable polymeric nanoparticles: A co-surfactant study. Asian J Pharm Sci..

[34] Cohen-Sela E, Chorny M, Koroukhov N, Danenberg HD, Golomb G (2009). A new double emulsion solvent diffusion technique for encapsulating hydrophilic molecules in PLGA nanoparticles. J Control Release..

[35] Chronopoulou L (2013). Chitosan-coated PLGA nanoparticles: a sustained drug release strategy for cell cultures. Colloids Surf B Biointerfaces.

[36] Pandit, J., Sultana, Y. & Aqil. M. Chitosan-Coated PLGA Nanoparticles of Bevacizumab as Novel Drug Delivery to Target Retina: Optimization, Characterization, and *in Vitro* Toxicity Evaluation. *Artif Cells Nanomed Biotechnol*. 1–11, 10.1080/21691401.2016.1243545 (2016). 10.1080/21691401.2016.1243545 27855494

[37] Zhao K (2014). Chitosan-coated poly(lactic-co-glycolic) acid nanoparticles as an efficient delivery system for Newcastle disease virus DNA vaccine. Int J Nanomed..

[38] Babu A (2014). Chitosan coated poly(lactic acid) polymeric nanoparticle-mediated combinatorial delivery of cisplatin and siRNA/plasmid DNA chemosensitizes cisplatin-resistant human ovarian cancer cells. Mol. Pharm..

[39] Lankveld DP (2011). Blood clearance and tissue distribution of PEGylated and non-PEGylated gold nanorods after intravenous administration in rats. Nanomedicine (Lond)..

[40] Prapainop K, Witter DP, Wentworth P (2012). A chemical approach for cell-specific targeting of nanomaterials: small-molecule-initiated misfolding of nanoparticle corona proteins. J Am Chem Soc..

[41] Mahmoudi M (2015). Crucial role of the protein corona for the specific targeting of nanoparticles. Nanomedicine (Lond)..

[42] Cheng J (2007). Formulation of functionalized PLGA-PEG nanoparticles for *in vivo* targeted drug delivery. Biomaterials.

[43] Su Y (2017). Paclitaxel-loaded star-shaped copolymer nanoparticles for enhanced malignant melanoma chemotherapy against multidrug resistance. Drug Des Devel Ther..

[44] Makadia HK, Siegel SJ (2011). Poly Lactic-co-Glycolic Acid (PLGA) as Biodegradable Controlled Drug Delivery Carrier. Polymers (Basel)..

[45] Alexis F (2005). Factors affecting the degradation and drug-release mechanism of poly(lactic acid) and poly[(lactic acid)-co-(glycolic acid)]. Polym. Int..

[46] Danhier F (2009). Paclitaxel-loaded PEGylated PLGA-based nanoparticles: *In vitro* and *in vivo* evaluation. J Control Release..

[47] Modi S, Anderson BD (2013). Determination of Drug Release Kinetics from Nanoparticles: Overcoming Pitfalls of the Dynamic Dialysis Method. Mol. Pharmaceutics.

[48] Zhang Z, Feng SS (2006). The drug encapsulation efficiency, *in vitro* drug release, cellular uptake and cytotoxicity of paclitaxel-loaded poly(lactide)-tocopheryl polyethylene glycol succinate nanoparticles. Biomaterials.

[49] Jin C (2009). Cytotoxicity of paclitaxel incorporated in PLGA nanoparticles on hypoxic human tumor cells. Pharm Res..

[50] Elias DR, Poloukhtine A, Popik V, Tsourkas A (2013). Effect of ligand density, receptor density, and nanoparticle size on cell targeting. Nanomedicine..

[51] Suzuki S (1993). Alterations of integrin expression in human lung cancer. Jpn J Cancer Res..

[52] Zhan C (2010). Cyclic RGD conjugated poly(ethylene glycol)-co-poly(lactic acid) micelle enhances paclitaxel anti-glioblastoma effect. J Control Release..

[53] Fröhlich E (2012). The role of surface charge in cellular uptake and cytotoxicity of medical nanoparticles. Int. J. Nanomedicine..

[54] Yu B, Zhang Y, Zheng W, Fan C, Chen T (2012). Positive Surface Charge Enhances Selective Cellular Uptake and Anticancer Efficacy of Selenium Nanoparticles. Inorg. Chem.

[55] Vilos C (2013). Paclitaxel-PHBV nanoparticles and their toxicity to endometrial and primary ovarian cancer cells. Biomaterials..

[56] Das GC, Holiday D, Gallardo R, Haas C (2001). Taxol-induced cell cycle arrest and apoptosis: dose-response relationship in lung cancer cells of different wild-type p53 status and under isogenic condition. Cancer Lett..

[57] Li R, Moudgil T, Ross HJ, Hu HM (2005). Apoptosis of non-small-cell lung cancer cell lines after paclitaxel treatment involves the BH3-only proapoptotic protein Bim. Cell Death Differ..

[58] Druškovič M, Šuput D, Milisav I (2006). Overexpression of Caspase-9 Triggers Its Activation and Apoptosis *in Vitro*. Croat Med J..

[59] Lee HH (2014). Combination treatment with paclitaxel and doxorubicin inhibits growth of human esophageal squamous cancer cells by inactivation of Akt. Oncol Rep..

[60] Ajabnoor GM, Crook T, Coley HM (2012). Paclitaxel resistance is associated with switch from apoptotic to autophagic cell death in MCF-7 breast cancer cells. Cell Death Dis..

[61] Luo X, Kraus WL (2012). On PAR with PARP: cellular stress signaling through poly(ADP-ribose) and PARP-1. Genes Dev..

[62] Mitchison TJ (2012). The proliferation rate paradox in antimitotic chemotherapy. Mol. Biol. Cell..

[63] Payne, S. & Miles, D. Mechanisms of Anticancer Drugs, In Gleeson, M. (Ed.), Scott-Brown’s Otorhinolaryngology: Head and Neck Surgery 7Ed. CRC Press, 34–46. 10.1201/b15118-6 (2008).

[64] Ikeda M (2010). DNA damage detected with gammaH2AX in endometrioid adenocarcinoma cell lines. Int J Oncol..

[65] Olive PL, Banath JP (2009). Kinetics of H2AX phosphorylation after exposure to cisplatin. Cytometry Part B..

[66] Mehta M (2016). HuR silencing elicits oxidative stress and DNA damage and sensitizes human triple-negative breast cancer cells to radiotherapy. Oncotarget..

[67] Schrump DS (2002). Pharmacokinetics of paclitaxel administered by hyperthermic retrograde isolated lung perfusion techniques. J Thorac Cardiovasc Surg..

[68] Lee JG, Wu R (2012). Combination erlotinib-cisplatin and Atg3-mediated autophagy in erlotinib resistant lung cancer. PLoS One..

[69] Matsuoka H, Furusawa M, Tomoda H, Seo Y (2008). Difference in cytotoxicity of paclitaxel against neoplastic and normal cells. Anticancer Res..

[70] Keum C-G (2011). Practical preparation procedures for docetaxel-loaded nanoparticles using polylactic acid-co-glycolic acid. Int J Nanomed.

[71] Patil Y, Panyam J (2009). Polymeric Nanoparticles for siRNA Delivery and Gene Silencing. Int J Pharm..

[72] Basotra M, Singh SK, Gulati M (2013). Development and Validation of a Simple and Sensitive Spectrometric Method for Estimation of Cisplatin Hydrochloride in Tablet Dosage Forms: Application to Dissolution Studies. ISRN Anal Chem..

[73] Ramesh R (2003). Melanoma differentiation-associated gene 7/interleukin (IL)-24 is a novel ligand that regulates angiogenesis via the IL-22 receptor. Cancer Res..

[74] Zeng R, Chen Y, Zhao S, Cui GH (2012). Autophagy counteracts apoptosis in human multiple myeloma cells exposed to oridonin *in vitro* via regulating intracellular ROS and SIRT1. Acta Pharmacol. Sin..

[75] Srivastava A (2016). Nanosomes carrying doxorubicin exhibit potent anticancer activity against human lung cancer cells. Sci Rep..

