# Supplementary material for: Chemodrug delivery using integrin-targeted PLGA-Chitosan nanoparticle for lung cancer therapy
Source: Sci Rep. 2017 Nov 7;7:14674. doi: 10.1038/s41598-017-15012-5 (PMC5676784; doi:10.1038/s41598-017-15012-5)
Supplement: Supplementary file 1 — Supplementary Data [file 41598_2017_15012_MOESM1_ESM.pdf]

Chemodrug delivery using integrin-targeted Chitosan-PLGA nanoparticle for lung cancer therapy

Anish Babu[1,6], Narsireddy Amreddy[1,6] , Ranganayaki Muralidharan[1,6], Gopal Pathuri[2,5], Hariprasad Gali[2,6], Allshine Chen[3,6], Yan D. Zhao[3,6], Anupama Munshi[4,6] , Rajagopal Ramesh[1,6,7*]

Department of [1]Pathology, [2]Pharmaceutical Sciences, [3]Biostatistics and Epidemiology, [4]Radiation Oncology, and [5]Medicine, The University of Oklahoma Health Sciences Center, Oklahoma City, Oklahoma 73104, USA; [6]Stephenson Cancer Center, The University of Oklahoma Health Sciences Center, Oklahoma City, Oklahoma 73104, USA; [7]Graduate Program in Biomedical Sciences, The University of Oklahoma Health Sciences Center, Oklahoma City, Oklahoma 73104, USA.

***Address for Correspondence**. Rajagopal Ramesh, Ph.D., Department of Pathology, Stanton L. Young Biomedical Research Center, Suite 1403, 975 NE, 10th Street, The University of Oklahoma Health Sciences Center, Oklahoma City, OK 73104, USA. Phone: 405-271-6101; E-mail: rajagopal-ramesh@ouhsc.edu

SUPPLEMENTARY MATERIAL

Table ST1. Quantification (normalized to actin) of protein bands in western blots of Figure 1

	**H1299**	**A549**	**H460**	**HCC827**	**H1437**	**H1975**	**MRC9**	**CCD16**	**WI38**
**Integrin αv**	**0.27**	**0.69**	**0.19**	**0.19**	**0.38**	**0.51**	**1.14**	**0.68**	**0.66**
**Integrin β3**	**0.55**	**0.16**	**0.32**	**0.46**	**0.33**	**1.21**	**1.16**	**0.85**	**0.69**

Table ST2. Particle size measurement of PTX-PLGA-CSNP-RGD formulations in the presence of different serum concentrations and for three different time points (1h, 6h and 24h).

Serum(%) used for NP incubation		Incubation Time (1h)	Incubation Time (6h)	Incubation Time (24h)
0	Size (nm)	225.25	224.06	229.38
	Zeta Potential (mV)	22.24	24.17	19.70
	PDI	0.085	0.025	0.017
2	Size (nm)	226.17	233.70	255.91
	Zeta Potential (mV)	5.27	1.94	-5.71
	PDI	0.069	0.194	0.144
5	Size (nm)	233.92	238.92	261.16
	Zeta Potential (mV)	0.44	-11.02	-15.48
	PDI	0.108	0.133	0.140
10	Size (nm)	250.29	266.85	305.85
	Zeta Potential (mV)	-17.88	-21.11	-22.94
	PDI	0.141	0.213	0.148

Table ST3. Quantification (fold changes compared to untreated control normalized to actin) of protein bands in western blots of Figures 6B and S9B.

				C-PARP	CASP9
**H1299**	**6 h**	**PTX-PLGA-CSNP-RGD**	**Fold Change**	4.39	3.43
		**PTX-PLGA-CSNP-RGD + RGD**		1.69	1.06
	**24 h**	**PTX-PLGA-CSNP-RGD**		14.22	7.18
		**PTX-PLGA-CSNP-RGD + RGD**		2.26	1.42
**A549**	**6 h**	**PTX-PLGA-CSNP-RGD**	**Fold Change**	1.42	2.14
		**PTX-PLGA-CSNP-RGD + RGD**		0.48	1.19
	**24 h**	**PTX-PLGA-CSNP-RGD**		3.36	5.89
		**PTX-PLGA-CSNP-RGD + RGD**		1.06	1.81
**H1975**	**6 h**	**PTX-PLGA-CSNP-RGD**	**Fold Change**	4.59	2.25
		**PTX-PLGA-CSNP-RGD + RGD**		2.41	1.92
	**24 h**	**PTX-PLGA-CSNP-RGD**		10.43	2.01
		**PTX-PLGA-CSNP-RGD + RGD**		5.00	1.88

Table ST4. Quantification (fold changes compared to untreated control normalized to actin) of protein bands in western blots of Figure S10B.

				Integrin α_V	C-PARP	CASP9
**H1299**	**24 h**	**PTX-PLGA-CSNP**	**Fold Change**	1.04	1.02	1.11
		**PTX-PLGA-CSNP-RGD**		1.13	1.41	1.01
	**48 h**	**PTX-PLGA-CSNP**		0.51	2.84	0.99
		**PTX-PLGA-CSNP-RGD**		0.41	4.18	0.94
**A549**				**Integrin α_V**	**C-PARP**	**CASP9**
	**24 h**	**PTX-PLGA-CSNP**	**Fold Change**	0.89	3.35	1.54
		**PTX-PLGA-CSNP-RGD**		0.43	5.18	2.06
	**48 h**	**PTX-PLGA-CSNP**		0.54	4.61	1.02
		**PTX-PLGA-CSNP-RGD**		0.19	8.75	1.34
**H1975**				**Integrin α_V**	**C-PARP**	**CASP9**
	**24 h**	**PTX-PLGA-CSNP**	**Fold Change**	0.88	3.17	1.54
		**PTX-PLGA-CSNP-RGD**		0.30	5.18	2.06
	**48 h**	**PTX-PLGA-CSNP**		0.54	4.61	1.02
		**PTX-PLGA-CSNP-RGD**		0.19	8.75	1.34
**MRC-9**				**Integrin α_V**	**C-PARP**	**CASP9**
	**24 h**	**PTX-PLGA-CSNP**		0.95	1.13	1.03
		**PTX-PLGA-CSNP-RGD**		0.88	1.44	1.04
	**48 h**	**PTX-PLGA-CSNP**		0.69	1.60	1.01
		**PTX-PLGA-CSNP-RGD**		0.69	2.44	1.14

Table ST5. Quantification (fold changes compared to untreated control normalized to actin) of protein bands in western blots of Figure 7B.

				C-PARP	CASP9	pH2AX
**H1975**	**24 h**	**CDDP-PLGA-CSNP**	**Fold Change**	2.35	4.08	20.91
		**CDDP-PLGA-CSNP-RGD**		8.75	8.59	21.49
	**48 h**	**CDDP-PLGA-CSNP**		2.75	2.23	20.93
		**CDDP-PLGA-CSNP-RGD**		4.19	27.25	38.29
**MRC-9**	**24 h**	**CDDP-PLGA-CSNP**	**Fold Change**	1.04	1.01	1.04
		**CDDP-PLGA-CSNP-RGD**		1.06	1.03	1.21
	**48 h**	**CDDP-PLGA-CSNP**		3.12	0.92	2.22
		**CDDP-PLGA-CSNP-RGD**		3.06	0.41	2.98

Table ST6. Quantification (fold changes compared to untreated control normalized to actin) of protein bands in western blots of Figure 8C.

				C-PARP	CASP9	
**NHBE**	**24 h**	**PTX**	**Fold Change**	1.20	1.03	
		**PTX-PLGA-CSNP**		1.07	0.98	
		**PTX-PLGA-CSNP-RGD**		1.15	0.98	
	**48 h**	**PTX**		2.13	2.54	
		**PTX-PLGA-CSNP**		0.82	2.19	
		**PTX-PLGA-CSNP-RGD**		1.63	1.16	
				**C-PARP**	**CASP9**	**pH2AX**
	**24 h**	**CDDP**	**Fold Change**	1.03	0.99	1.05
		**CDDP-PLGA-CSNP**		0.96	1.06	1.01
		**CDDP-PLGA-CSNP-RGD**		1.55	0.95	1.07
	**48 h**	**CDDP**		1.07	1.05	0.82
		**CDDP-PLGA-CSNP**		1.11	1.07	1.05
		**CDDP-PLGA-CSNP-RGD**		1.12	1.05	0.62

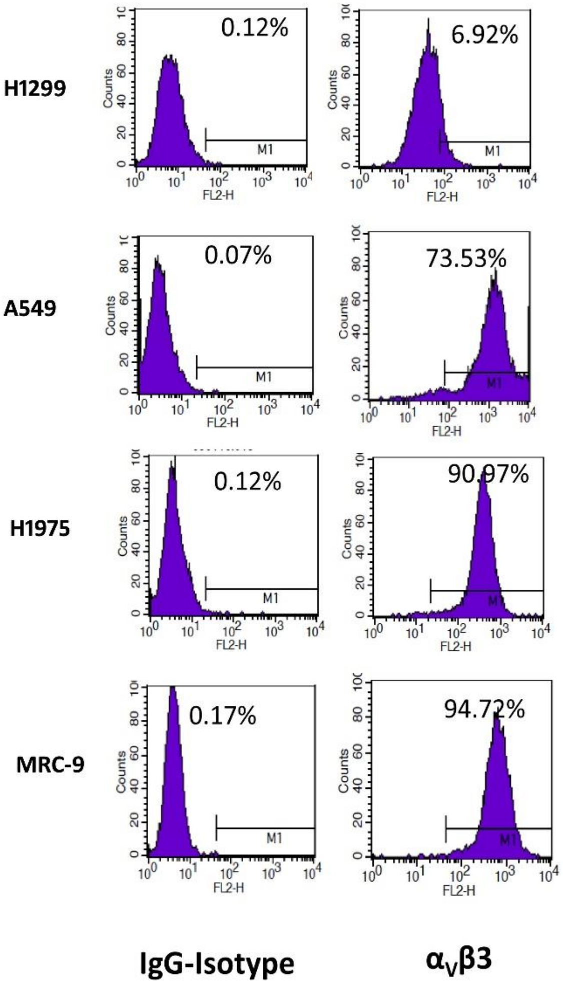

Figure S1. Flow cytometry data of Integrin $\alpha_v\beta_3$ baseline expression

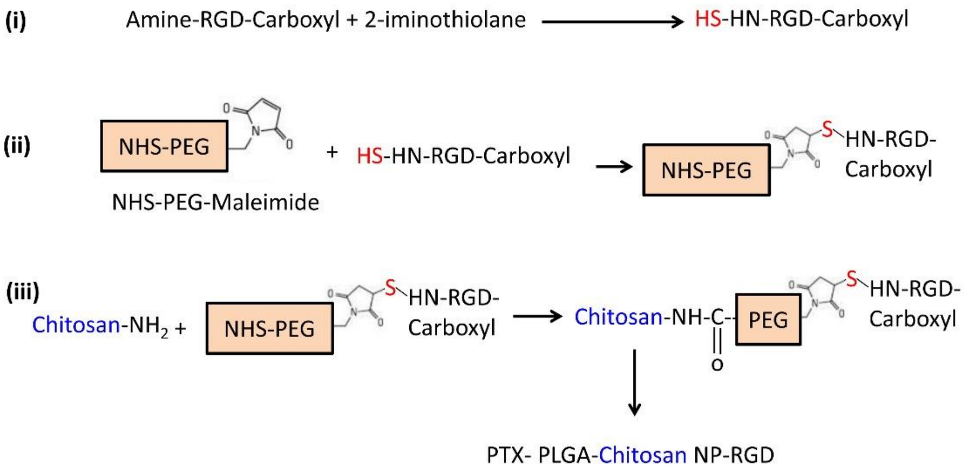

Figure S2. Scheme of PTX-Chitosan NP-RGD synthesis

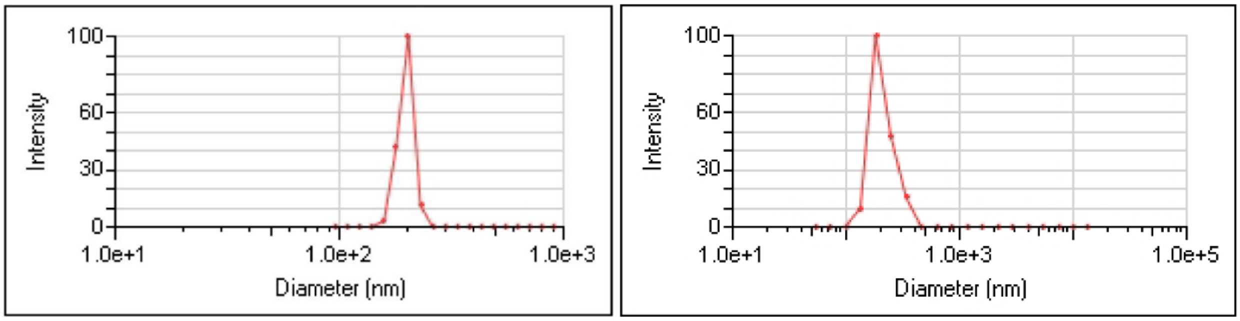

Diameter (nm) 199.76

Day 1 : PTX-PLGA-CSNP-RGD

Diameter (nm) 208.81

Day 14 : PTX-PLGA-CSNP-RGD

Figure S3. Dynamic light scattering measurement of PTX-PLGA-CSNP-RGD particle sizes at day 1 and 14 after preparation. Particles were kept in 4^{0}C in aqueous solution, showing its stability for 2 weeks.

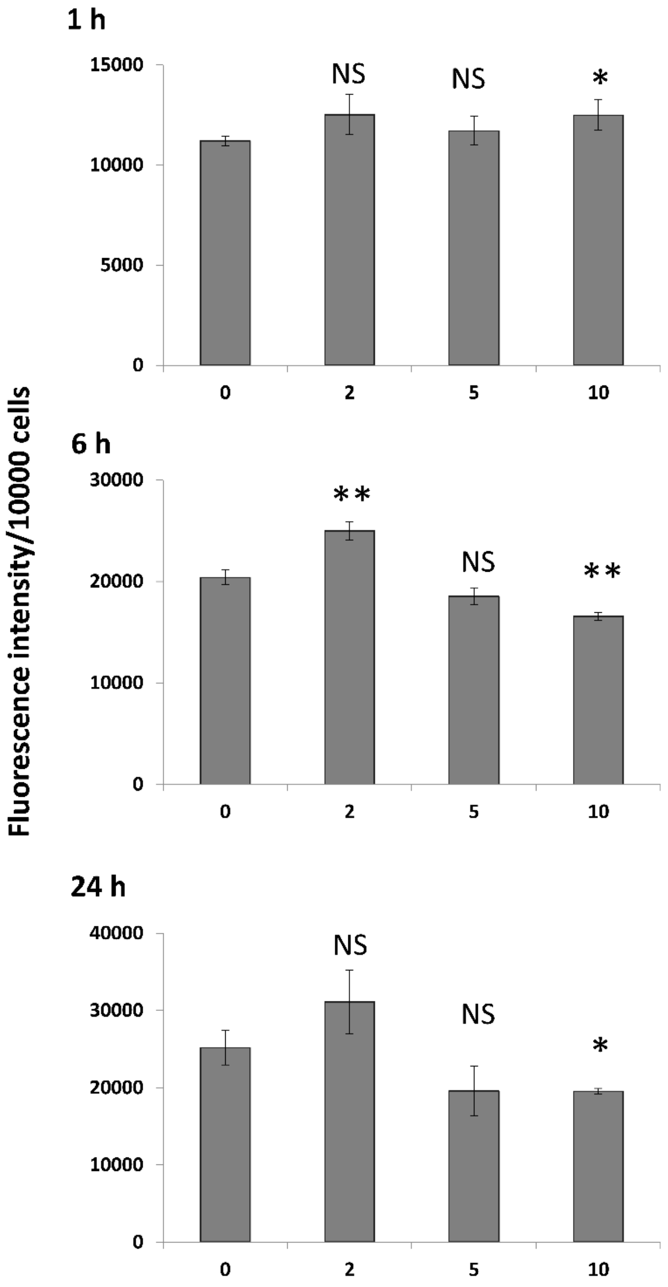

Figure S4. Uptake of 0, 2, 5 or 10 % serum incubated FluTax-PLGA-CSNP-RGD in A549 cells. The nanoparticles were incubated with serum (of 0, 2, 5 or 10 %) for 1h, 6h or 24h prior to addition in the cell culture in serum free media. Further cells were harvested at 1h, 6h or 24h, washed 3 times in PBS and measured the FluTax fluorescence in cells using Envision plate reader using FITC filter. The fluorescence intensity was then normalized to 10000 cells and plotted. *$p<0.05$; **$p<0.01$; NS, non-significant

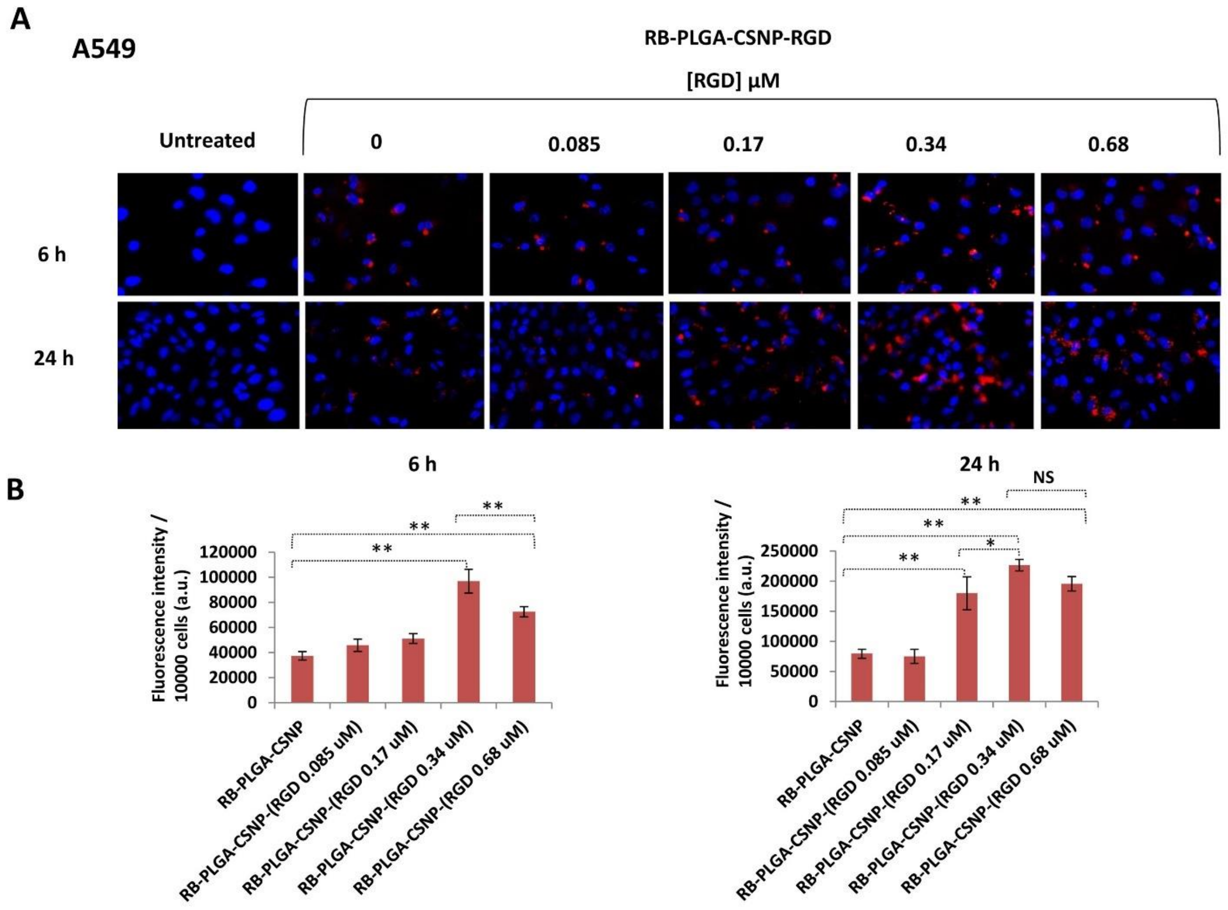

Figure S5. RGD Optimization. (A) Microscopy images (B) and quantitative measurement of A549 cells treated with RB-PLFA-CSNP-RGD. RGD concentration in nanoparticle was varied from 0 to 0.68 µM. **p*<0.05; ***p*<0.01; NS, non-significant

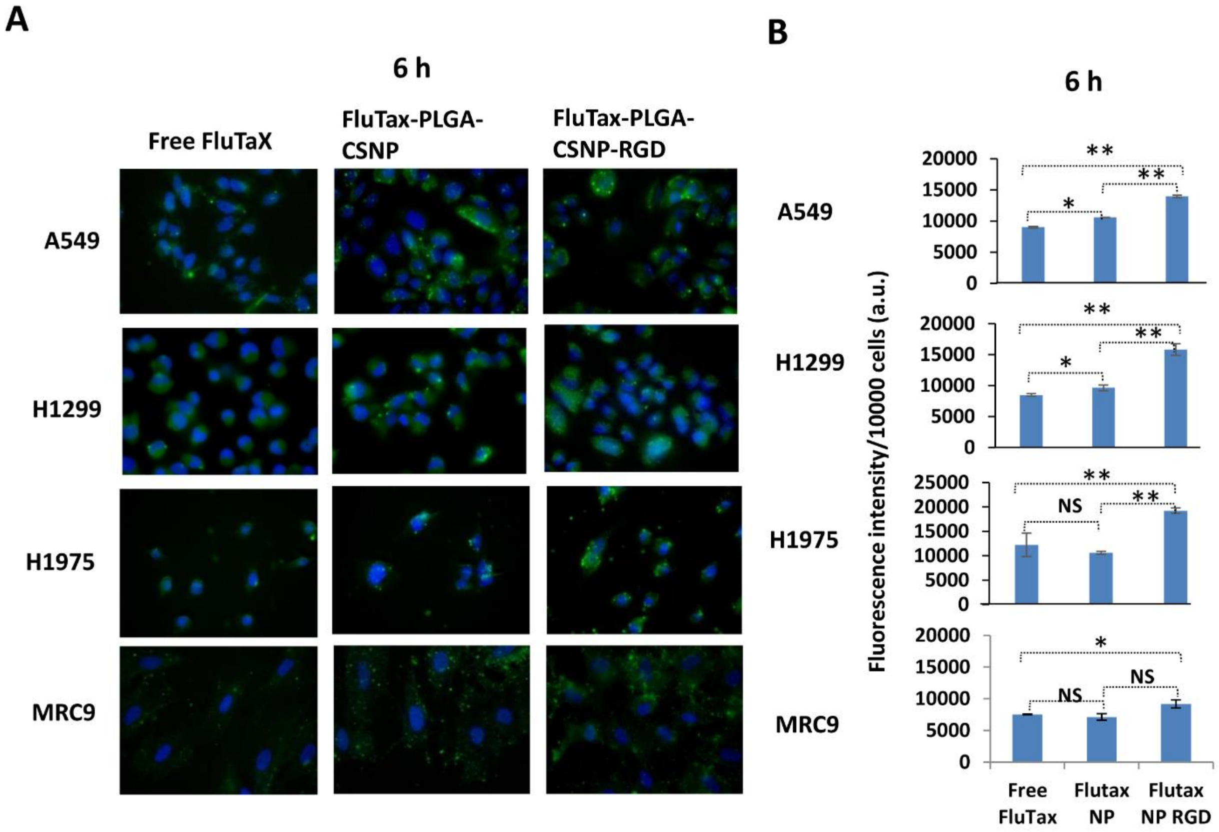

Figure S6. Cell uptake studies in lung cancer (A549, H1299, and H1975) and lung fibroblast (MRC9) cell lines. (A) Microscopy images and, (B) quantitative representation of cells treated with fluorescent paclitaxel (FluTax) either in free form, FluTAX-PLGA-CSNP and FluTax-PLGA-CSNP-RGD for 6 hours. *p<0.05; **p<0.01; NS, non-significant

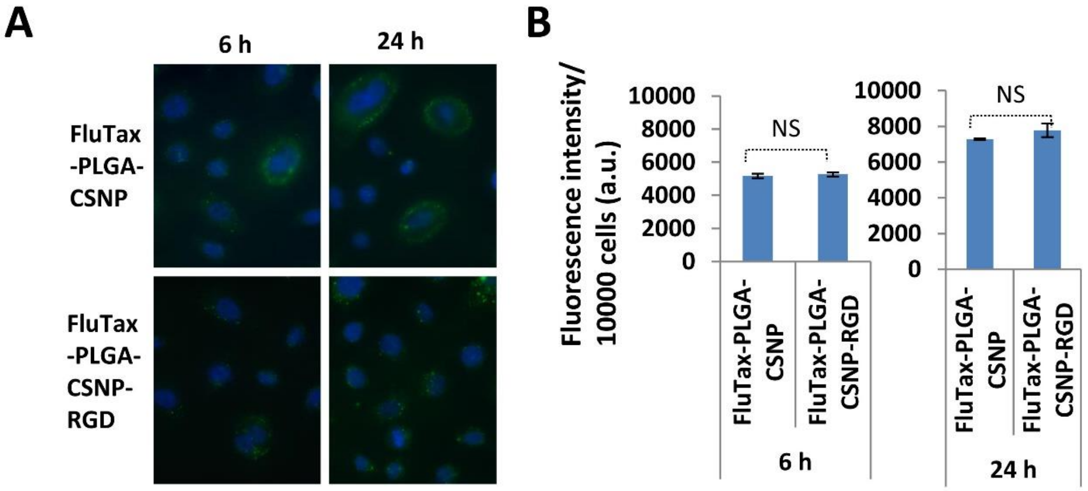

Figure S7. (A) Fluorescent microscopic images and (B) Bar graph depicting fluorescence intensity/10000 cells in NHBE cell lines after 6 h and 24 h post incubation with of FluTax-PLGA-CSNP or FluTax-PLGA-CSNP-RGD. NS, non-significant

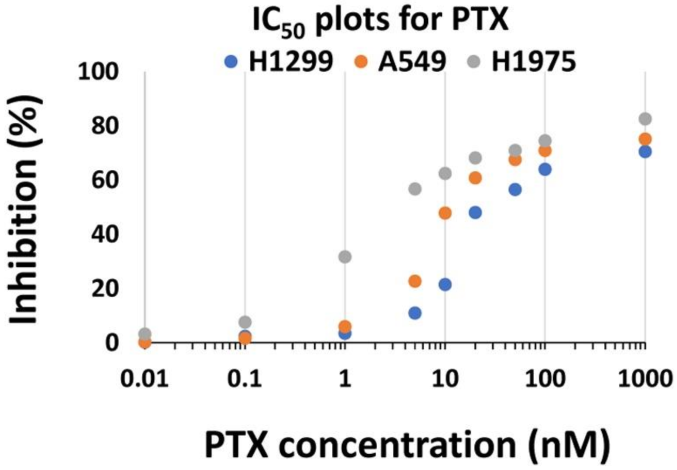

Figure S8. Plot for IC50 determination for paclitaxel in lung cancer cell lines

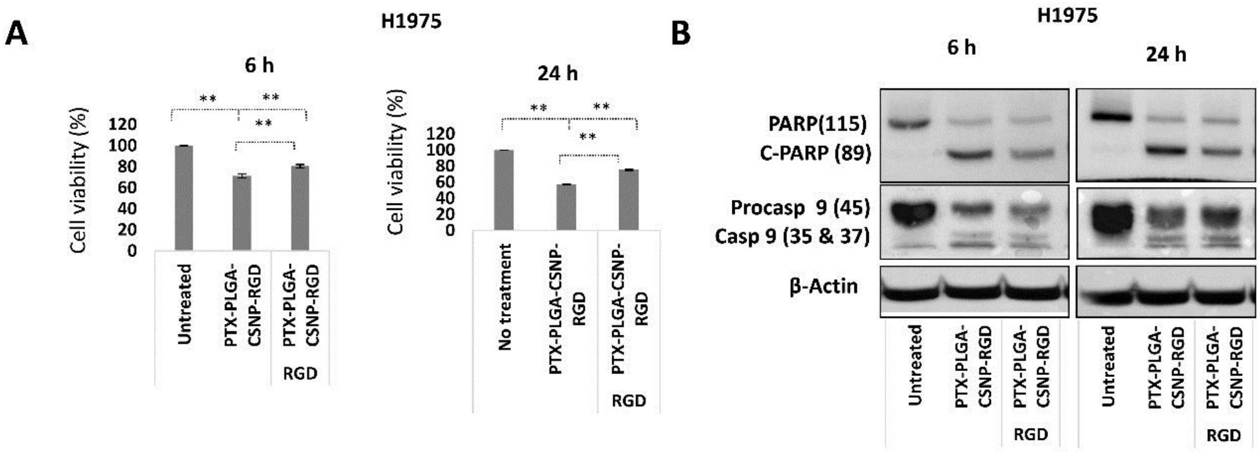

Figure S9. Integrin blocking study in H1975 cells. (A) Cell viability showed pretreatment with free RGD abrogated PTX-PLGA-CSNP-RGD cytotoxicity. (B) Western blotting showed cleaved caspase 9 and cleaved PARP activity was reduced in cells pretreated with free RGD prior to PTX-PLGA-CSNP-RGD treatment. *p<0.05; **p<0.01; NS, non-significant

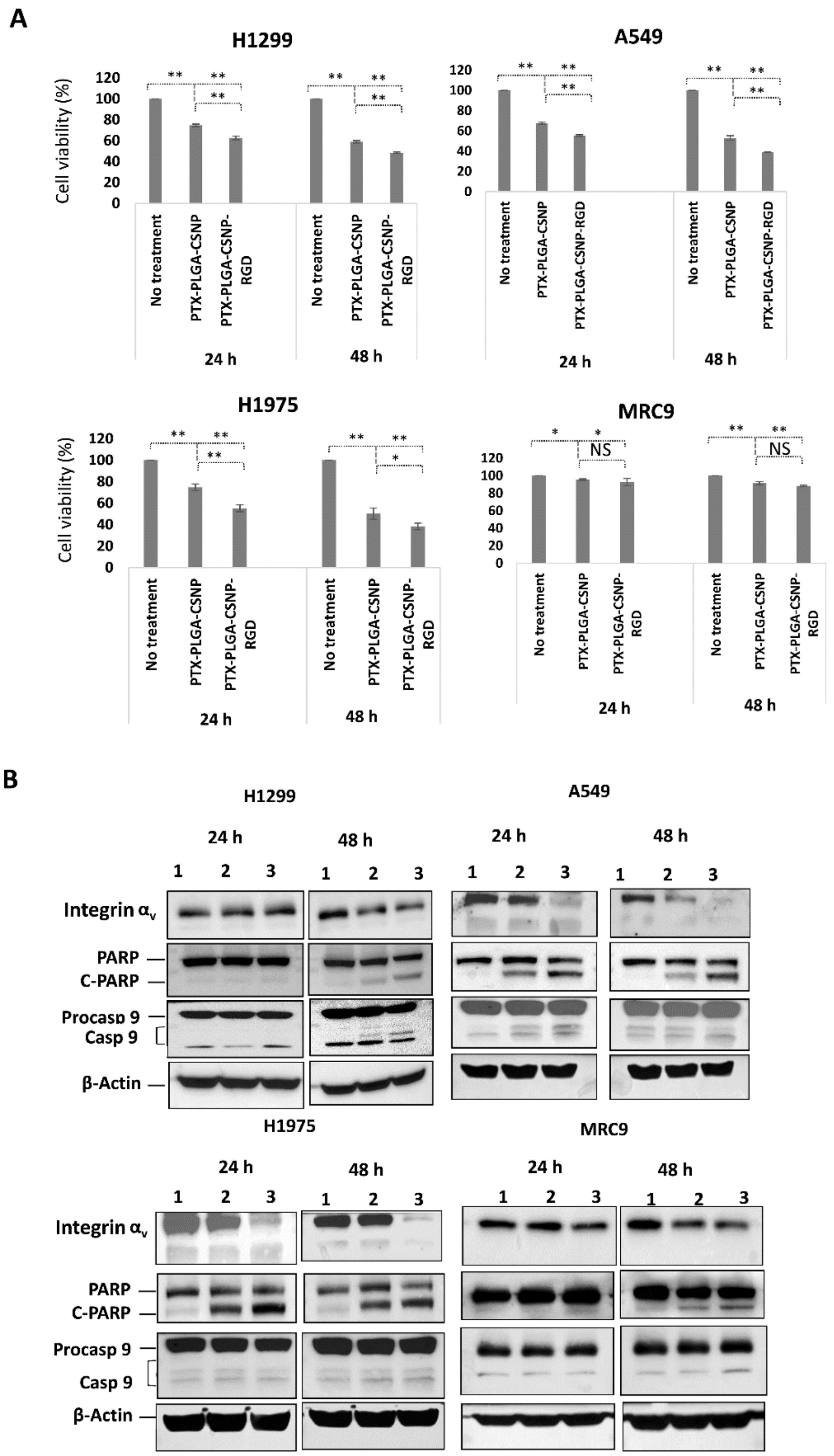

1. Untreated
2. PTX-PLGA-CSNP
3. PTX-PLGA-CSNP-RGD

Figure S10. (A) Cell viability and (B) western blot analysis apoptotic proteins of lung cancer cells and normal lung fibroblasts when treated with PTX-PLGA-CSNP or PTX-PLGA-CSNP-RGD compared to untreated controls. *p<0.05; **p<0.01; NS, non-significant

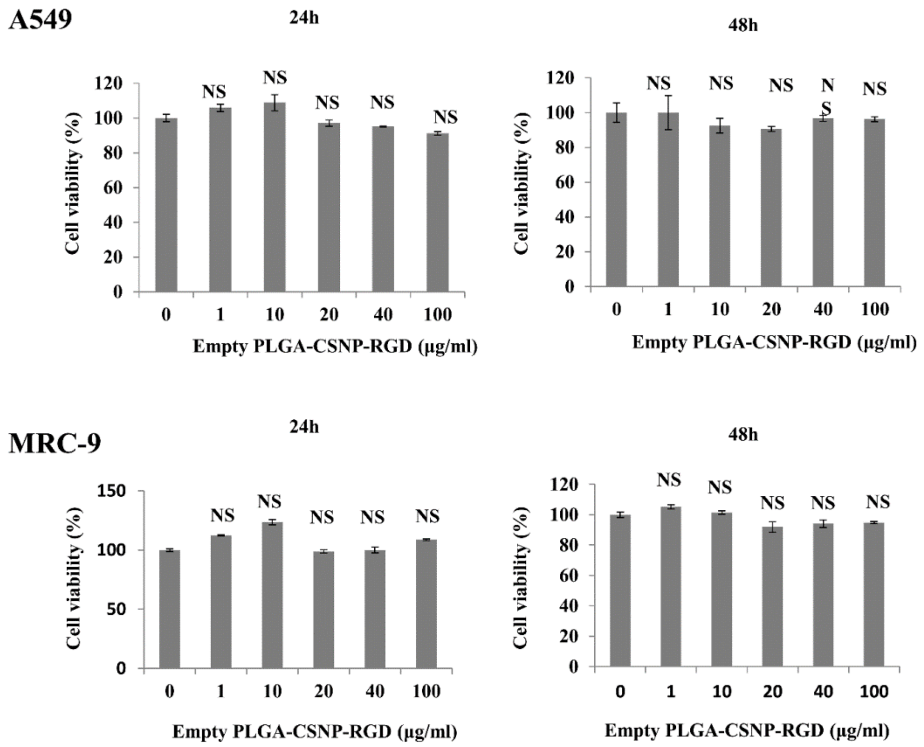

Figure S11. A549 and MRC-9 cell viability at 24h, and 48h after treatment with various concentrations (0-100 μg/mL) of PLGA-CSNP-RGD (empty nanoparticles). n=3, p<0.05, NS: non-significant.

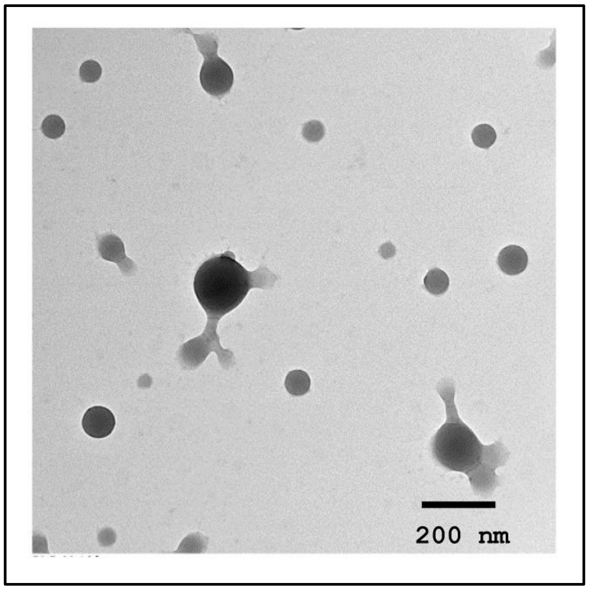

Figure S12. TEM image of CDDP-PLGA-CSNP-RGD

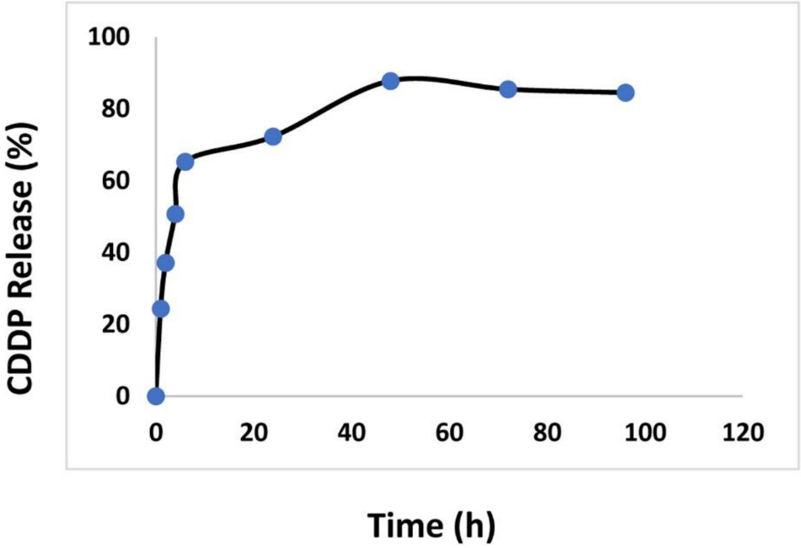

Figure S13. *In vitro* release profile of CDDP from PLGA-CSNP-RGD in PBS (pH 7.2).

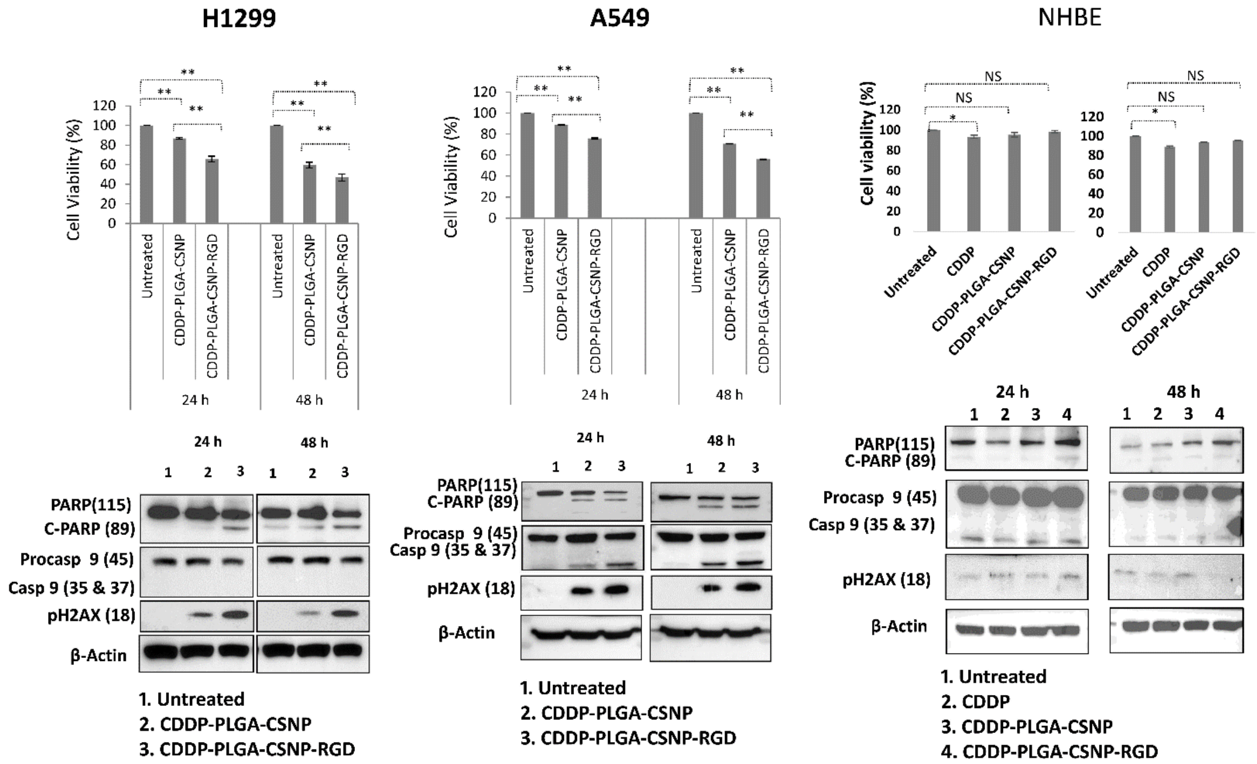

Figure S14. Cell viability and western bot analysis of H1299 (40 µM CDDP), A549 (33 µM CDDP) and NHBE (40 µM CDDP) cells when treated with CDDP nanoparticle formulations. **p*<0.05; ***p*<0.01; NS, non-significant
